# Bimetallic Co–Fe sulfide and phosphide as efficient electrode materials for overall water splitting and supercapacitor

**DOI:** 10.1186/s11671-023-03837-1

**Published:** 2023-04-04

**Authors:** Shiva Bhardwaj, Rishabh Srivastava, Teddy Mageto, Mahesh Chaudhari, Anuj Kumar, Jolaikha Sultana, Sanjay R. Mishra, Felio Perez, Ram K. Gupta

**Affiliations:** 1 Department of Physics, Pittsburg State University, Pittsburg, KS 66762 USA; 2 National Institute of Material Advancement, Pittsburg State University, Pittsburg, KS 66762 USA; 3 Department of Physics and Materials Science, The University of Memphis, Memphis, TN 38152 USA; 4 Integrated Microscopy Center, The University of Memphis, Memphis, TN 38152 USA; 5 Nano-Technology Research Laboratory, Department of Chemistry, GLA University, Mathura, Uttar Pradesh 281406 India; 6 Department of Chemistry, Pittsburg State University, Pittsburg, KS 66762 USA

**Keywords:** Nanocomposites, Electrocatalysts, Supercapacitor, Cobalt-iron, Sulfide, Phosphorized

## Abstract

The major center of attraction in renewable energy technology is the designing of an efficient material for both electrocatalytic and supercapacitor (SC) applications. Herein, we report the simple hydrothermal method to synthesize cobalt-iron-based nanocomposites followed by sulfurization and phosphorization. The crystallinity of nanocomposites has been confirmed using X-ray diffraction, where crystalline nature improves from as-prepared to sulfurized to phosphorized. The as-synthesized CoFe-nanocomposite requires 263 mV overpotential for oxygen evolution reaction (OER) to reach a current density of 10 mA/cm^2^ whereas the phosphorized requires 240 mV to reach 10 mA/cm^2^. The hydrogen evolution reaction (HER) for CoFe-nanocomposite exhibits 208 mV overpotential at 10 mA/cm^2^. Moreover, the results improved after phosphorization showing 186 mV to reach 10 mA/cm^2^. The specific capacitance (C_sp_) of as-synthesized nanocomposite is 120 F/g at 1 A/g, along with a power density of 3752 W/kg and a maximum energy density of 4.3 Wh/kg. Furthermore, the phosphorized nanocomposite shows the best performance by exhibiting 252 F/g at 1 A/g and the highest power and energy density of 4.2 kW/kg and 10.1 Wh/kg. This shows that the results get improved more than twice. The 97% capacitance retention after 5000 cycles shows cyclic stability of phosphorized CoFe. Our research thus offers cost-effective and highly efficient material for energy production and storage applications.

**Supplementary Information:**

The online version contains supplementary material available at 10.1186/s11671-023-03837-1.

## Introduction

Energy is the main driver of humanity’s advancements technologically as well as socio-economically. The rapid advancement in these sectors, coupled with the significant increase in the global population, leads to increased demand to produce energy. It is also important to develop renewable energy sources to lower the global CO_2_ emissions produced by burning fossil fuels, which is the current primary source in various industries today. Electrocatalytic water splitting is one such renewable energy source that has attracted great attention in recent years [1–4]. Electrocatalytic water splitting involves hydrogen evolution reaction (HER) and oxygen evolution reaction (OER), both of which offer promising outcomes in terms of renewable energy production. However, due to the intermittent nature of this energy source, it is imperative to develop electrochemical energy storage devices. On the other hand, batteries and supercapacitors (SCs) are the two main electrochemical energy storage devices extensively studied today [5–8]. SCs are of great interest owing to their rapid charge/discharge, long cycle stability, and high-power density. However, due to the utilization of noble materials as electrodes, the further application of SCs faces a critical limitation. To this end, further research efforts have been applied to developing and fabricating electrode materials that are both high-performance and low-cost. Such materials include sulfides, transition metal oxides, phosphides, nitrides, oxalates [9–11], etc. However, even with this benefit, these materials still exhibit high overpotentials and low specific capacitance values compared to the noble materials currently utilized [12].

In this context, developing nanostructured materials such as nanocomposites is an important area of research that has garnered increasing interest in recent years. Compared to conventional materials for batteries and SCs, nanomaterials offer greatly improved ionic transport and electronic conductivity [13]. Additionally, these materials facilitate the provision of high specific capacities through the occupation of all available intercalation sites available in the particle volume. As a result, these materials can improve electrocatalytic water-splitting performance. In a study conducted by Gao et al*.* [14] for example, composite electrodes comprising Cu foam and hierarchically Co–Fe-based catalyst particles were fabricated to offer channel-like structures for the transport of electrolytes and release of oxygen gas bubbles. The study reported high electrocatalytic OER performance, with the electrodes exhibiting a low overpotential of 293 mV at *j* = 50 mA/cm^2^. In another study by Pang et al*.* [15], hierarchical amorphous NiCoP with dandelion-like arrays were anchored on nanowires via hydrothermal and phosphorization processes and utilized to enhance overall water splitting. This unique hierarchical structure of the nanomaterial provides a large surface area for catalysis, thereby facilitating the provision of abundant active sites and low mass and charge transfer resistance. Additionally, the structure is maintained after 10 h of electrocatalysis thereby exhibiting excellent mechanical stability. As a result, the study reported a low OER overpotential of 276 mV to attain 10 mA/cm^2^.

Despite the efficiency and environmental friendliness of electrocatalytic water splitting in producing hydrogen, the processes of water reduction and oxidation at the electrodes suffer from sluggish kinetics caused by the high overpotential of OER at the anode [16]. This inhibits the utilization of electrocatalytic water splitting for large-scale hydrogen production. Therefore, the development of an electrocatalyst with enhanced catalytic activity is the cornerstone for the improvement of the efficiency of the electrocatalytic water-splitting reactions [17]. To this end, transition metals such as Ni, Co, and Fe have been extensively studied for utilization as electrocatalysts for OER [18–20]. In general, transition metal-based electrocatalysts possess a more impressive intrinsic activity and stability in alkaline media than their noble-based counterparts at a fraction of the cost. Additionally, the adjustable chemical reactivity, excellent corrosion resistance, and theoretically high thermodynamic stability and efficiency of Ni and Co make these materials even more promising for electrocatalytic applications [21].

Furthermore, bimetallic transition metal electrodes such as NiCo and CoFe exhibit higher electrochemical activity toward OER than monometallic electrodes due to modification of the local electronic structure. Due to their robust electrocatalytic capability, especially regarding electrocatalytic activity for water oxidation [22], CoFe electrocatalysts have seen a rise in research interest in recent years. Typically, CoFe electrocatalytic materials utilized for OER include CoFe include CoFe hydroxides, CoFe LDHs, CoFe oxides, and CoFe alloy composites. Nanostructured CoFe electrode materials can further enhance catalytic activity as they provide high specific surface area, abundant active sites, and excellent conductivity [23]. To further enhance electrical conductivity, hetero-atom dopants such as phosphide and sulfide can be incorporated into the CoFe bimetallic electrode. For instance, Mendoza-Garcia et. al. utilized phosphorization of Co–Fe–O nanocubes to fabricate sea urchin-like (Co_x_Fe_1−x_)_2_P nanostructures as active catalysts for OER [24], with an optimum overpotential of 370 mV at 10 mA/cm^2^ is achieved. The formation of a core–shell CoFeP/CoFeO nanostructure is the main reason for this performance as the bimetallic phosphide core stabilizes the oxide shell leading to increased activity and stability of the catalyst in alkaline media. In another study by Huang et al*.* [25], phosphorus-doped CoFeS hybrids were synthesized via a multi-element composition-engineering approach to developing a CoFeSP/CNT bifunctional electrocatalyst. When utilized as an oxygen evolution electrode, the CoFeSP/CNT exhibits excellent electrocatalytic performance with a low OER overpotential of 262 mV at a current density of 10 mA/cm^2^. The utilization of phosphorous as a doping material is the main driving factor for the improved electrochemical performance, as a phosphorus-free CoFeS/CNT oxygen evolution electrode exhibits a higher OER overpotential of 296 mV at a current density of 10 mA/cm^2^. Herein, we synthesized the Co–Fe-based nanocomposite using the hydrothermal synthesis route, followed by the sulfurization (Co–Fe–S) and phosphorization (Co–Fe–P) process. The synthesis route requires only an hour at low temperature (< 100 °C) to prepare the nanocomposite which is comparatively faster, than any other hydrothermal method reported by researchers. Moreover, the agglomerated 3-D dense spherical type of morphology was obtained in Co–Fe–P which further improves the SC results more than twice as of Co–Fe-nanocomposite. Also, the electrocatalytic activity shows significant improvement in results after phosphorization. The development of a high-performance material for use in electrocatalytic and SC applications has become the focal point of renewable energy research and development.

## Experimental section

### Materials and methods

For the synthesis of cobalt-iron-based nanocomposite cobalt nitrate hexahydrate (Co(NO_3_)_2_·H_2_O, Strem Chemicals), iron-nitrate nonahydrate (Fe(NO_3_)_2_·H_2_O, Strem Chemicals), sodium hydroxide (NaOH), sodium sulfide (Na_2_S), sodium phosphite (Na_3_PO_4_) ethanol (C_2_H_5_OH), and deionized (DI) H_2_O were purchased from Fisher Scientific, USA.

#### Synthesis of CoFe-nanocomposite

The CoFe-nanocomposite was synthesized using 3 mmol Co(NO_3_)_2_·H_2_O as the cobalt source, whereas 2 mmol Fe(NO_3_)_2_·H_2_O was used as an iron source. These two compounds were added to 200 ml of DI-H_2_O and allowed to form a clear solution. Furthermore, the pH of the obtained solution was maintained at ⁓13 by adding 3 M NaOH (8 ml) solution. The above solution was then transferred to a Teflon-lined autoclave at 80 °C for 60 min. Finally, the obtained nanocomposite was centrifuged and washed several times with ethanol to remove impurities. Subsequently, the CoFe-nanocomposite was dried at 70 °C overnight. The CoFe-nanocomposite was drawn into a very fine powder using a hand-mortar pistil and then used for characterization.

#### Synthesis of CoFe-S-nanocomposite

The above-obtained powder was then sulfurized using Na_2_S in a ratio of 1:10 (CoFe: Na_2_S) and further transferred to a Teflon-lined autoclave at 140 °C for 24 h. The obtained nanocomposite was centrifuged and washed several times with ethanol to remove impurities. Subsequently, the CoFe-S-nanocomposite was dried at 70 °C overnight. The CoFe-S-nanocomposite was drawn into a very fine powder using a hand-mortar pistil and then used for characterization.

#### Synthesis of CoFe-P-nanocomposite

**Fig. 1:**
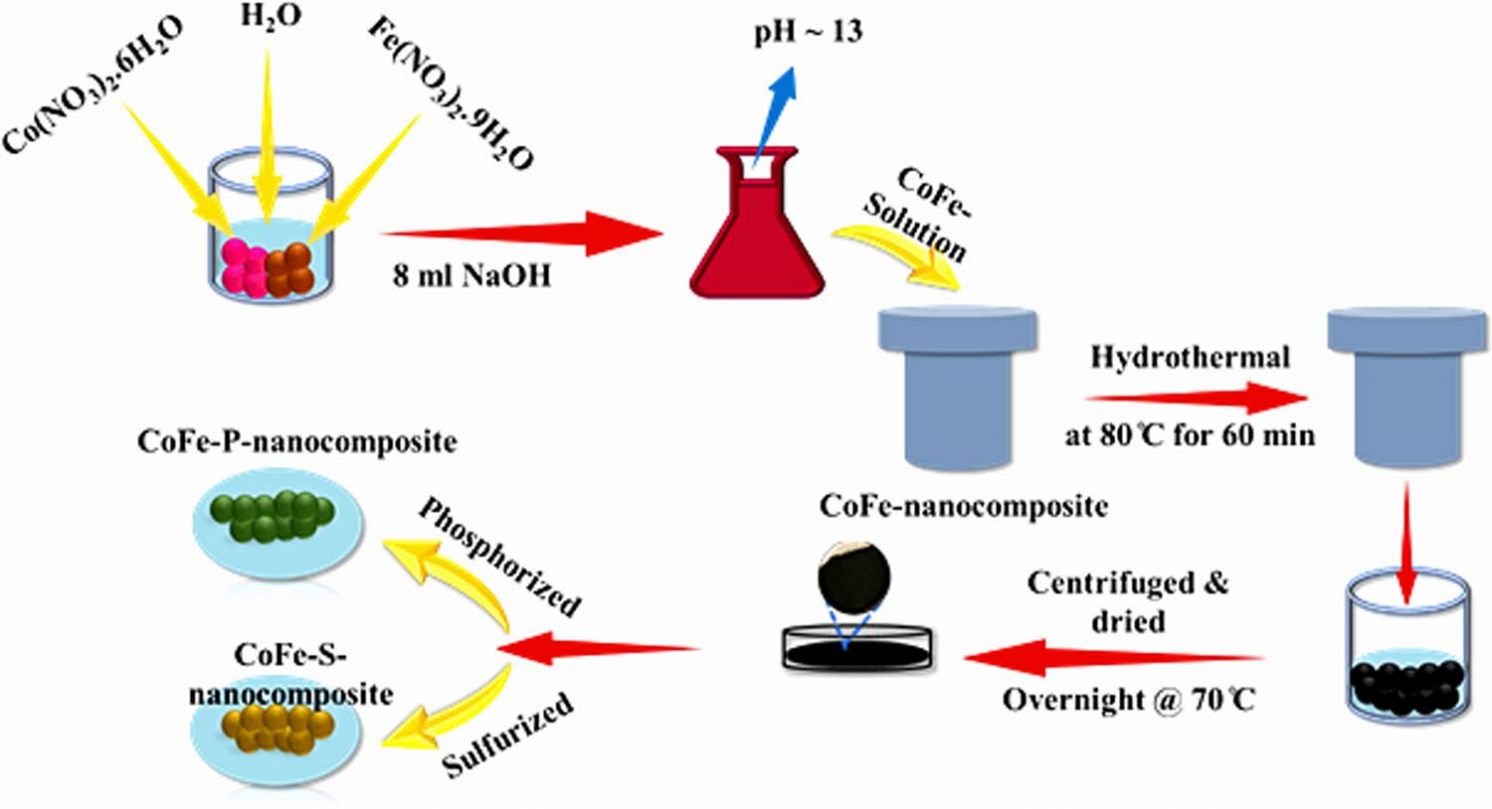
Schematic illustration of the synthesis of CoFe, CoFe-S, and CoFe-P-nanocomposite

The above-obtained powder was then phosphorized using Na_3_PO_4_ in a specific ratio using a tube furnace at 320 °C for 5 h at 5 °C/min from room temperature. The CoFe-P-nanocomposite was allowed to cool down to room temperature and then used for characterization. The schematic illustration is shown in Fig. 1 for all the nanocomposites.

### Instrumentation

#### Materials characterization

The crystal structure of prepared nanocomposites was characterized by X-ray diffraction (XRD) using Shimadzu X-ray diffractometer with Cu Kα (λ = 1.5406 Å) radiation was used to record the XRD patterns in 2θ mode with angular range 10°–80°. The surface morphology of the CoFe, CoFe-S, and CoFe-P nanocomposite was analyzed by a scanning electron microscope (SEM) (at 10 keV using Phenom, Oak Park, CA, USA). The X-ray photoelectron spectrometer (Thermo Scientific K-alpha, XPS) equipped with a monochromatic X-ray source at 1486.6 eV, corresponding to the Al K-alpha line, was used to perform XPS measurements on all the samples.

#### Electrochemical study

The electrochemical measurements were carried out with a Versa stat 4–500 workstation (Princeton Applied Research, TN, USA) at room temperature using three electrode configurations. To study the electrocatalytic activity Ag/AgCl (sat. KCl) was used as a reference electrode whereas, for supercapacitive studies, Hg/HgO was used as a reference electrode. For both systems, a platinum wire was used as a counter electrode. All the electrodes were prepared using a standard ratio of 8:1:1 of synthesized material, poly-vinyl-fluoride (PVDF), and n-methyl polypropylene (NMP) to form a slurry. The prepared slurry was spread uniformly on Ni-foam via dipping and then transferred to the vacuum dried at 70 °C for 48 h. 1 M and 3 M KOH solution was for electrocatalytic and supercapactive studies respectively. Linear sweep voltammogram (LSV) was recorded at a fixed slow scan rate of 2 mV/s to minimize the capacitive current. All potentials were converted to a reversible hydrogen electrode (RHE) scale using the following equation E_RHE_ = E_Ag/AgCl_ + E^o^_Ag/AgCl_ + 0.059 pH. The operating voltage window for SC studies was 0–0.6 V.

## Results and discussion

### Structural characterization

**Fig. 2:**
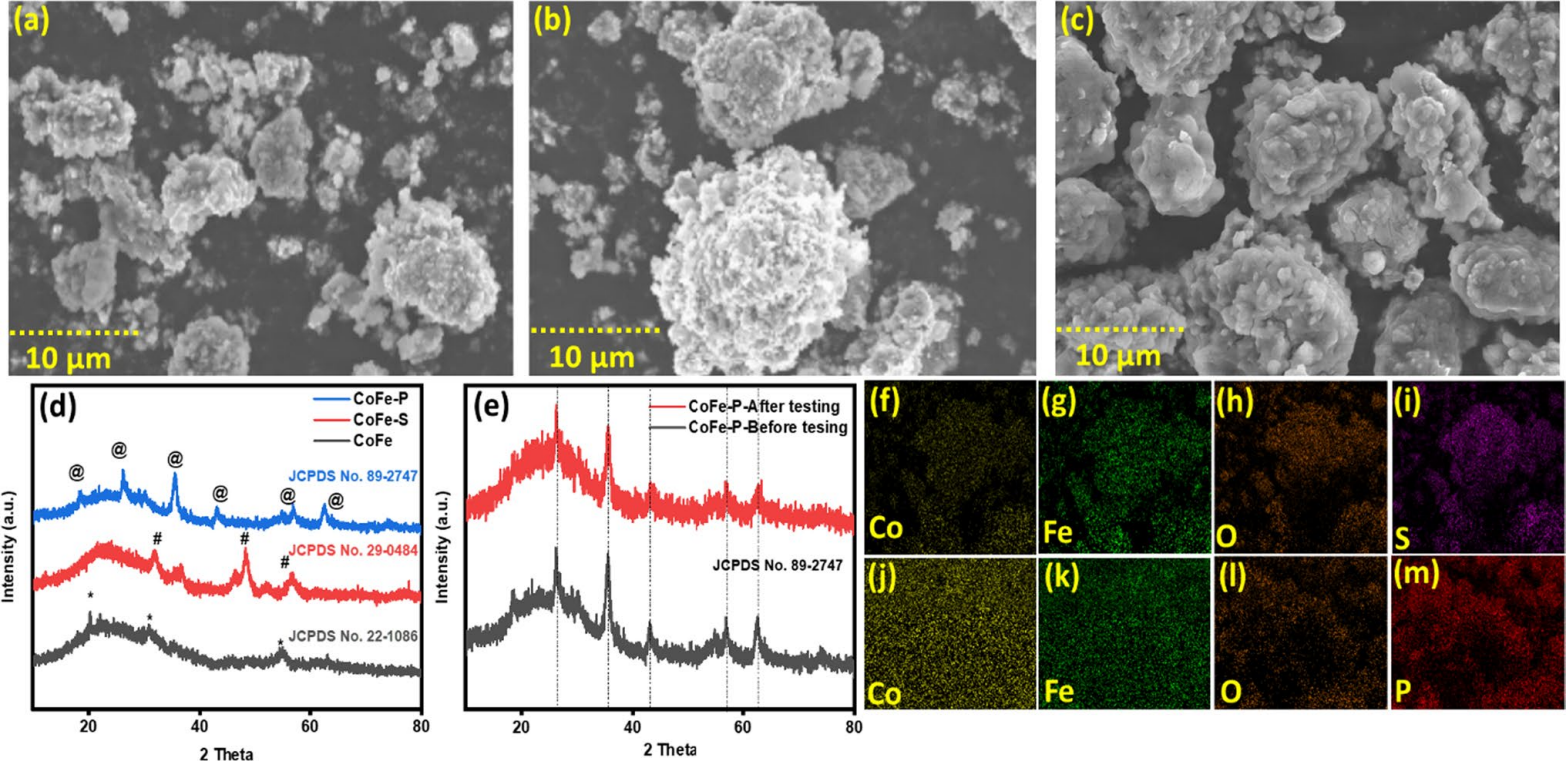
**a**–**c** SEM images of CoFe, CoFe-S, and CoFe-P-nanocomposite at 10 µm. **d** Shows the XRD to depict the crystalline nature of nanocomposites. **e** depicts the XRD pattern of CoFe-P before and after the electrochemical testing. **f**–**i** is the elemental mapping images of CoFe-S. **j**–**m** Shows the elemental confirmation for CoFe-P

The globular morphology of nanocomposites was discovered during scanning electron microscopy (SEM). Figure 2a shows the irregular globular structure of CoFe-nanocomposite, providing a better surface area for the catalyst and electrolyte to adsorb into its surface. The irregular globular structure converts broccoli like globular during the formation of CoFe-S-nanocomposite providing more surface area to the catalyst and electrolyte to get adsorbed into higher quantity on its surface. A slight increase in the particle size has been observed in going from CoFe to CoFe-S which might be due to the porous structure as shown in Fig. 2b. CoFe-P-nanocomposite structure gets agglomerated and densely populated while going from CoFe to CoFe-P allowing CoFe-P to possess high surface area for better specific capacitance. The as shown in Fig. 2c which provides better adsorption and desorption of ions into its surface [26].

Furthermore, the synthesized nanocomposites were tested using X-ray diffraction (XRD) to understand structural properties. The crystallinity of nanocomposites was confirmed through the peaks shown in Fig. 2d. The peaks at 2θ = 20.26°, 30° and 55° marked as * represent the formation of CoFe_2_O_4_ with JCPDS 22–1086 [27] while the peaks at 31.8°, 48.3° marked as # correspond to the (222), and (511) planes of Co_8_FeS_8_ JCPDS 29-0484, [28] whereas the peak at and 56° corresponds to the CoFe_2_O_4_. The peaks in CoFe-P marked as @ at 20°, and 56.6° are due to the presence of CoFe_2_O_4_, whereas the peaks at 35.4°, and 42.9° in CoFe-P correspond to the (111), and (211) planes of Co_1_Fe_0.1_P with JCPDS 89-2747 [29]. The peaks marked as @ at 26° and 62° correspond to orthorhombic Co_2_P with planes (211), (422) with JCPDS no 29-0497 [30–32] represent the mixed phase of CoFe-P [33]. Moreover, the stability of crystalline nature of CoFe-P before and after electrochemical test in alkaline medium, as shown in Fig. 2e, was confirmed through all the matched peaks, where peak intensity decreases slightly but does not affect the crystalline nature of CoFe-P. To better understand the distribution of P and S elemental mapping has been performed where the distribution of elements are shown in Fig. 2f–i for CoFe-S, whereas Fig. 2j–m comprehend the elements for CoFe-P.

Subsequently, X-ray photoelectron spectroscopy (XPS) was employed to confirm the synthesized nanocomposite's chemical states, binding energies, and elemental composition. The Fig. S1d–f exhibits the high-resolution spectrum of Co in CoFe, CoFe-S, and CoFe-P, confirming the 2p_1/2_ and 2p_3/2_ peaks, which correspond to the transition states of Co^2+^ ↔ Co^3+^ due to peaks at 797, 793.2, and 778.3 eV. The other two satellite peaks at 803.2 and 782.5 eV are assigned to the stretching vibration of Co^3+^ [34–36]. Furthermore, the unaltered structural morphology of nanocomposites was also evidenced as no shifts in XPS peaks for CoFe, confirming the stability of nanocomposites. The sharp peaks at 724.8 and 711.5 eV corresponds to Fe^2+^ 2p_1/2_ and Fe^2+^ 2p_3/2_ atoms taking part in the reaction [34, 37]. Moreover, the peak at 722 and 720 eV are the satellite peaks obtained due to the formation of vibrational bonds Fig. S1g–i [38]. The peak confirmed the doping of sulfur at 170.6 and 169.2 eV, indicating the involvement of S_2p_ atoms during the formation of CoFe-S also it corresponds to the S–O bond formation as shown Fig. S1j [39]. Moreover, the CoFe-P contains phosphorus peaks at 134.8 are satellite peaks and 133.6 eV shows the P-O bond on the surface supporting the presence of phosphorous P_2p_ atoms was depicted in Fig. S1k [40]. The XPS confirms the elemental atomic % of Co, Fe, and O, which is confirmed to be 1:2:4, forming the CoFe_2_O_4_ nanocomposite. The XPS survey of CoFe (Fig. S2a), CoFe-S (Fig. S2b), and CoFe-P (Fig. S2c) shows the existence of corresponding elements. The oxygen vacancies and composite’s O^2−^ ions presence was confirmed through peaks at 533.1–533.2 shows the chemically bonded O atoms to the surface, 532.4–533.5 due to the metal hydroxyl and some carbonate formation at the surface of nanocomposite, and 529–530.6 eV corresponds to the metal–oxygen (M–O) bond of O^1^s atoms depicting interfacial bonding between CoFe_2_O_4_ (Fig. S1a, d and g) [41, 42].

### Electrochemical studies

#### Electrocatalytic activity

**Fig. 3:**
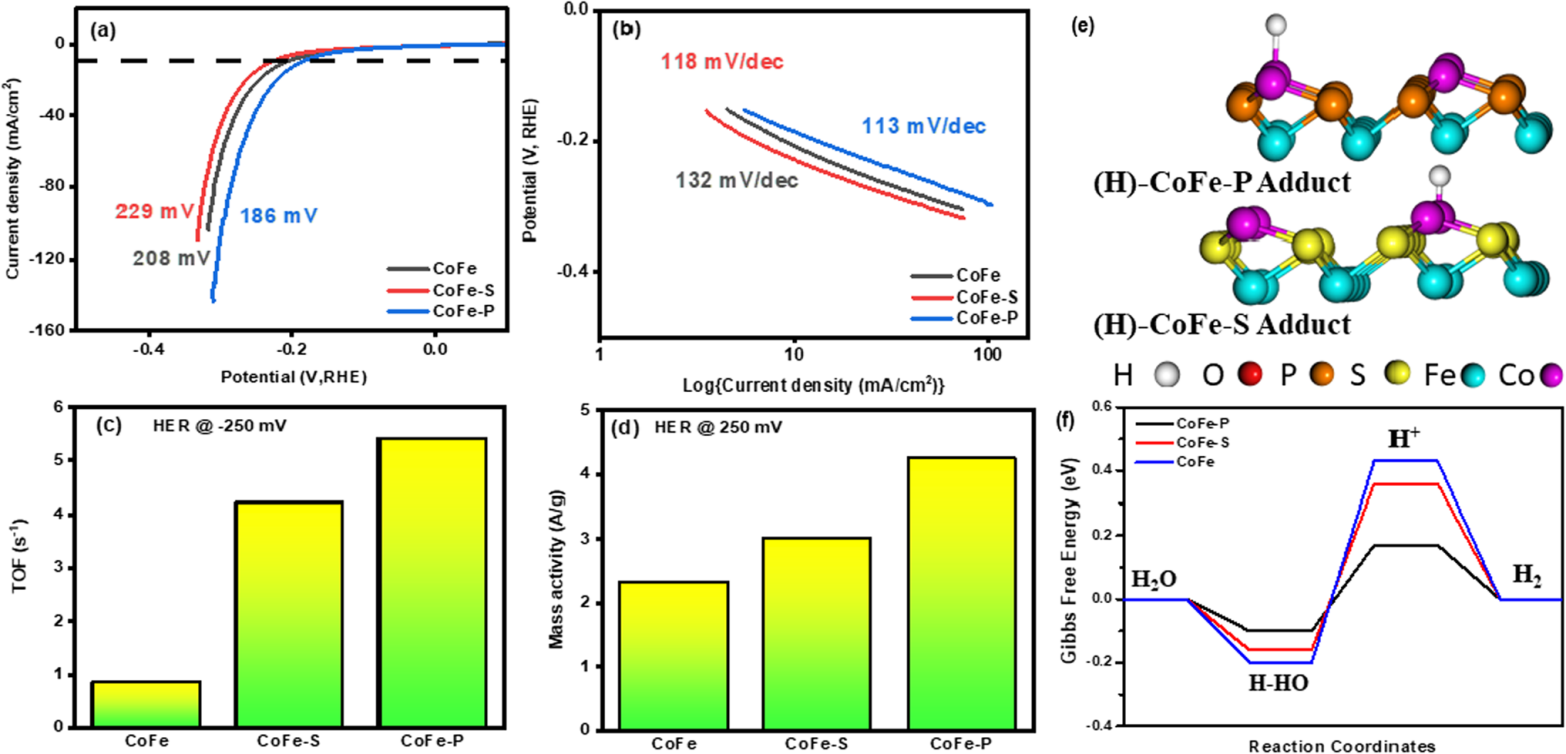
**a** LSV curves of the samples; comparison of overpotential required to drain a current density of 10 mA/cm^2^, **b** corresponding Tafel slopes of the samples, **c** TOF at an overpotential of 250 mV, **d** Mass activity at an overpotential of 250 mV, **e** Optimized H* state on Co-site of CoFe-S and CoFe-P samples, and **f** Gibbs free enrgy diagram for HER on Co-site of CoFe-S and CoFe-P samples

The HER performance of CoFe, CoFe-S, and CoFe-P was tested by taking out the LSV curve. The overpotential values of the as-prepared nanocomposites are demonstrated in Fig. 3a. The CoFe-P exhibited an overpotential of 186 mV at 10 mA/cm^2^ which is relatively smaller than the other samples. Besides, it was recorded that CoFe-S and CoFe required 19% and 10% less potential to attain a current density of 10 mA/cm^2^, respectively, indicating an inferior catalytic activity. The HER in alkaline media is determined under two steps i.e., Volmer and Heyrovsky [43]. The mechanism reactions are as given by Eqs. (1)–(2).

1 $${\text{Volmer Step}}:{\text{ H}}_{{2}} {\text{O}} + {\text{e}}^{ - } \leftrightarrow {\text{OH}}^{ - } + {\text{H}}_{{{\text{ad}}}}$$

2 $${\text{Heyrovsky Step}}:{\text{ H}}_{{2}} {\text{O}} + {\text{e}}^{ - } + {\text{H}}_{{{\text{ad}}}} \leftrightarrow {\text{OH}}^{ - } + {\text{H}}_{{2}}$$

The onset potential of HER is determined by the Volmer principle. Based on Fig. 3a, it is obvious to conclude that the CoFe-P on the NF greatly enhanced the water dissociation property at the Volmer step. However, the Heyrovsky step is the rate-limiting step for HER, therefore, the kinetics of the as-prepared composites was determined by the Tafel slopes, referred to as the rate-determining step in HER, were deduced from LSV curves with approximately 84.9% iR compensation (after evaluating impedance spectra at open circuit voltage), depending on the following Eq. (3).

3 $$\eta =a+ \frac{2.3RT}{\alpha nF}logj$$

where $\eta$ is the overpotential, α is the charge transfer coefficient, 2.3RT/αnF is the slope obtained from the plot drawn between potential and log *j,* which is named as Tafel slope, j stands for current density, R symbolizes gas constant, T is the temperature and n represents the number of electrons. The Tafel slopes are illustrated in Fig. 3b. In this sense, the Tafel slope corresponds to the charge transfer coefficient. Smaller the Tafel slope higher the charge transfer coefficient. Therefore, the CoFe-P addressed the lower kinetics with 113 mV/dec, which results in the swift transfer of electrons due to a more significant charge transfer coefficient, designating superior catalytic activity. The CoFe-P nanocomposite delineated the best HER activity than the other samples. The introduction of P did influence the catalytic attributes of the composite. Additionally, the Fe-rich microstructure of CoFe-P also enrich the morphology and showed a dense globular cloud, exhibiting a noticeable deterioration in overpotential. Further, the comprehensive kinetic behavior of the electrocatalyst was studied by another important parameter referred to as Turnover frequency (TOF) [44]. It determines the reaction rate or how rapidly a catalyst can turn reactants into products per unit time at a specific over potential of 250 mV. The given Eq. 4 was employed to evaluate the value of TOF.

4 $$TOF= \frac{j{N}_{a}}{nF\tau }$$

where N_a_ signifies the Avogadro number, n is the number of electrons transferred to emit a molecule of the product (in HER, for H_2_, it is 2), and τ is the surface concentration of active sites or number of participating atoms in the catalyst material. Figure 3c shows TOF, the CoFe-P achieved five times greater TOF than the CoFe. Similarly, CoFe-S displayed better kinetics than the CoFe as it has 4 times greater TOF, indicating excellent water-splitting activity. Sulfurization and phosphorization treatment can enhance the electrocatalytic activity of transitional metal-based compounds. For instance, Linghao et al*.* [32] reported that the incorporation of a lower electronegative element, P into the CoFe could tune the synergistic bonding between CoFe because of their optimum size. Moreover, P filling may improve the electrocatalytic activity, and facilitates the interaction between metal ions and lone pair-containing species to trap and operate as an active region. Therefore, in this case, the 3D dense globular cloud-like structure in CoFe-P created a larger surface area for the reaction to have happened and increased the water-splitting activity. Moreover, mass activity (MA) was accounted for in this study to understand the catalytic behavior of nanostructured materials. MA is the crucial tool represented in A/g and provides insight into the rough surface of the electrocatalyst when deposited mass on NF is considered to normalize its current density instead of the geometrical area of the deposited material on the NF taking part in the electrochemical testing [14]. The catalyst deposited on the NF might not have a planar or smooth surface. Therefore, Fig. 3d is calculated to report MA an intrinsic detail of the catalyst at a specific overpotential of 250 mV. This is defined by Eq. (5).

5 $$MA=\frac{{j}_{(\eta )}}{active\,mass\,loading}$$

The value of MA is 2.3208, 3.0081 and 4.2390 A/g for CoFe, CoFe-S, and CoFe-P, respectively. CoFe-P showed high performance of the catalyst, the surface area exposed to the electrolyte with hierarchically grown nanostructured arrays, resulting in high MA. Therefore, when expose to electrolytes, TOF and MA attributed information on the intrinsic properties of the electrocatalyst and their behavior. Table S1 contains the comprehensive study of as-prepared samples for HER performance on different parameters.

**Fig. 4:**
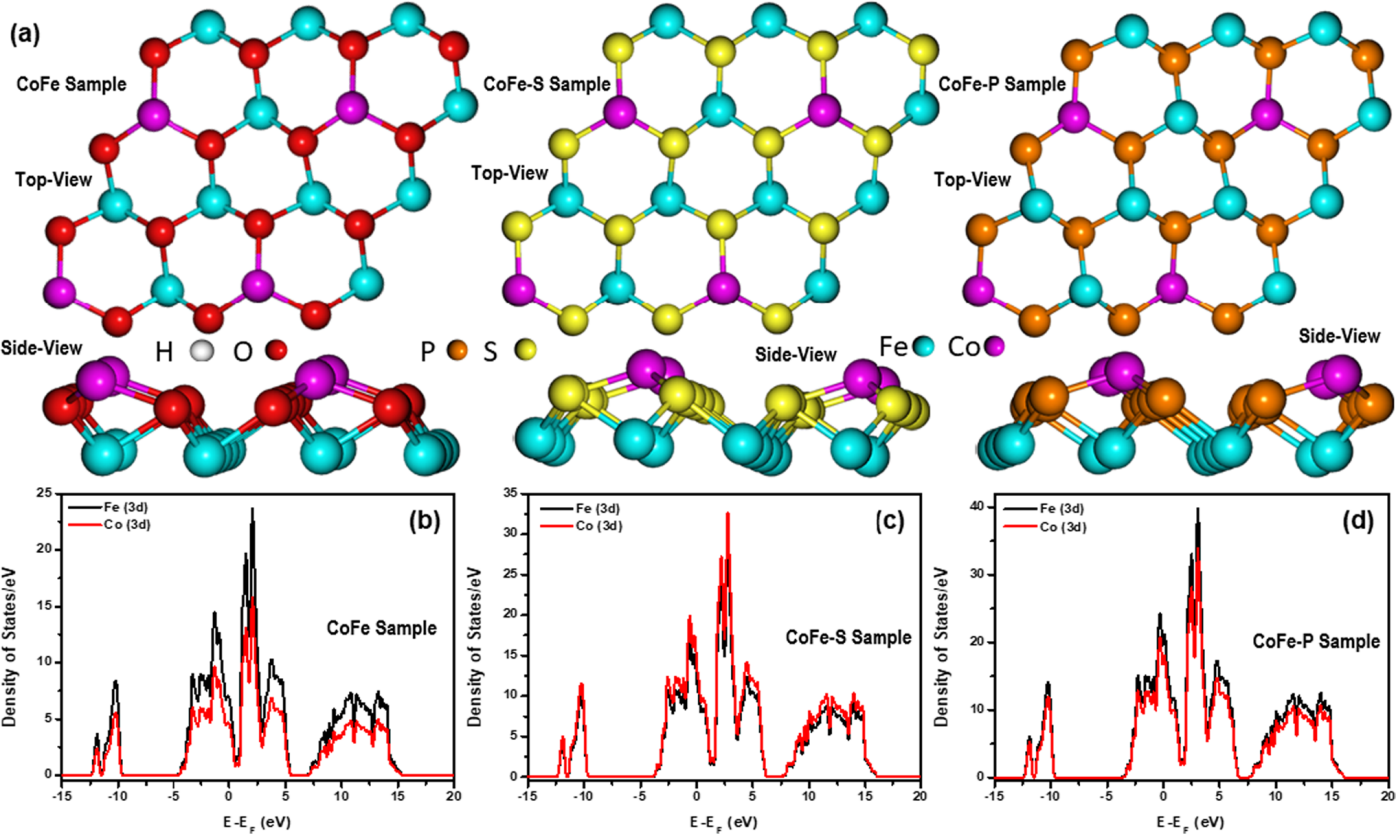
**a** Top and side views of the optimized geometries of CoFe, CoFe-S, and CoFe-P samples, and DOS of **b** CoFe, **c** CoFe-S, and **d** CoFe-P samples

In addition, because these samples have two active sites (Co and Fe) where catalytic reactions occur, DFT experiments were conducted to figure out the reaction mechanism and the true active site. The optimized geometries of CoFe, CoFe-S, and CoFe-P samples are shown in Fig. 4a. The chemical structure of each sample was constructed using the JCPDS information. As illustrated in Fig. 4b–d, the density of states (DOS) of each sample was analyzed to understand the chemistry of the change in the electronic structure of Co and Fe synergy via S- and P-atom inclusion. The results demonstrated that the P-atom in the CoFe-P sample could spread the electron density distribution over the fermi level of both the Co and Fe atoms much more than the S- and O-atoms in the CoFe-S and CoFe-O samples, which can be attributed to the P-lower atom's electronegativity and small size to adjust with the Co and Fe-sites. When compared to other samples, these facts may benefit the electrocatalytic activities of the CoFe-P sample.

Furthermore, to demonstrate the HER activity of CoFe, CoFe-S, and CoFe-P samples in alkaline media, the states H^+^, H* (* is active site) (Fig. 3e), and H_2_* were simulated, and the corresponding Gibbs free energies (ΔE_H*_) were calculated (Fig. 3f), taking both the Co and Fe as active sites into account [45, 46]. To obtain good HER activity, the ΔE_H*_ value is believed to be close to zero. When compared to the Fe-site, the first H_2_O binding on the co-site of each sample exhibited the lowest energy barriers for HER, especially for the CoFe-P sample. This remarkable HER on the CoFe-P sample can be attributed to the incorporation of P-atoms in the CoFe synergy, which significantly increased the DOS of Co (3d) as compared to Fe (3d), improving the conducive nature of the Co-site, thus lowering the HER energy barriers, resulting in good HER on the CoFe-P sample's Co-site [47].

Furthermore, the electrocatalytic OER is also recognized as a highly prized component of electrochemical activity during the water-splitting process. The OER takes place at the surface of active metal (M) and includes the following Eqs. (6)–(9).

6 $${\text{M}}^{*} + {\text{OH}}^{ - } \leftrightarrow {\text{M}}^{*} {\text{OH}} + {\text{e}}$$

7 $${\text{M}}^{*} {\text{OH}} + {\text{OH}}^{ - } \leftrightarrow {\text{M}}^{*} {\text{O}} + {\text{H}}_{{2}} {\text{O}} + {\text{e}}$$

8 $${\text{M}}^{*} {\text{O}} + {\text{OH}}^{ - } \leftrightarrow {\text{M}}^{*} {\text{OOH}} + {\text{e}}$$

9 $${\text{M}}^{*} {\text{OOH}} + {\text{OH}}^{ - } \leftrightarrow {\text{M}}^{*} + {\text{O}}_{{2}} + {\text{H}}_{{2}} {\text{O}} + {\text{e}}$$

**Fig. 5:**
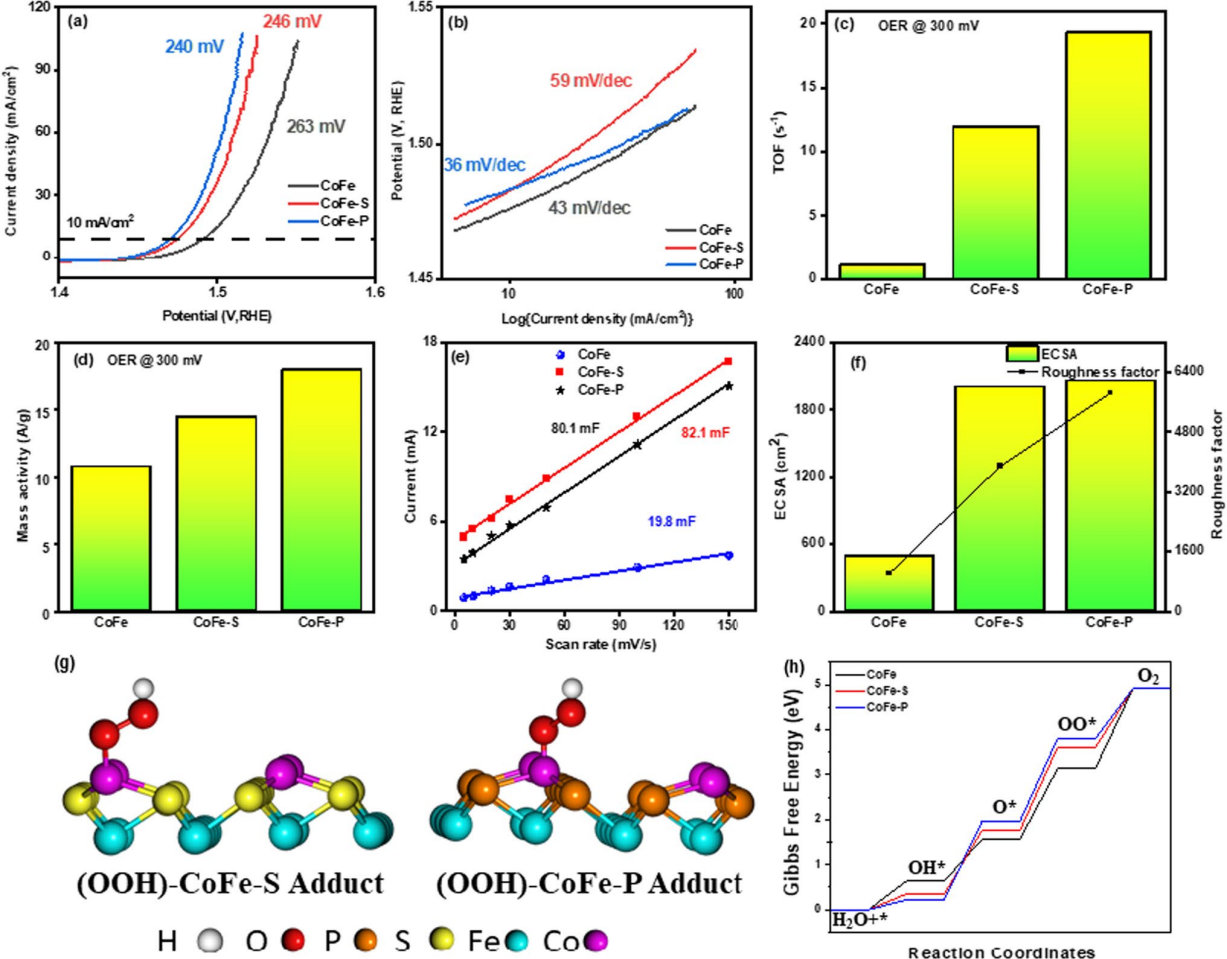
Electrochemical performance for OER: **a** LSV curves of the samples; comparison of overpotential required to drain a current density of 10 mA/cm^2^, **b** corresponding Tafel slopes of the samples, **c** TOF at an overpotential of 300 mV, **d** MA at an overpotential of 300 mV, **e** Double layer capacitance, **f** ECSA and RF values, **g** Optimized OOH* state (rate determining step) on Co-site of CoFe-S and CoFe-P samples, and **h** Gibbs free energy diagram for OER on Co-site of CoFe-S and CoFe-P samples

Figure 5a shows the OER polarization curve of the as-prepared samples. CoFe, CoFe-S, and CoFe-P needed an onset overpotential of 263, 246, and 240 mV to achieve a current density of 10 mA/cm^2^. In addition, the CoFe-S and CoFe-P have a Tafel slope of 59 mV/dec and 36 mV/dec, respectively, as shown in Fig. 5b, which corresponds that CoFe-P showed better kinetics due to a higher charge transfer coefficient. Hence, more electrons can flow through the electrode supporting O*, OH*, and OOH* adsorption. Further, the TOFs and MA were measured for all the samples to evaluate the intrinsic properties of the nanocomposite at a 300 mV overpotential. The values of TOF and MA are tabulated in Table S2. A linear increase in the value of TOFs and MA was noticed. The TOF (for OER, n = 4) value of CoFe-S was 10 times greater than CoFe. On the other hand, the CoFe-P was approximately 20 times better than CoFe as shown in Fig. 5c and d. Similarly, roughness exercised significant results for MA. Hence, the MA of CoFe-P is 17.955 A/g, indicating the best sample for O_2_ and H_2_ production. Moreover, this signifies that CoFe-P exhibits an adsorbate evolution mechanism (AEM) which shows the active metal cation act as a binder to OH^−^ ions into the solution. Zhang et al*.* [48] and his group performed OER activity of CoFeP nanocages which demonstrated highly dense hollow structure morphology with more exposed active sites, further influencing the deterioration in the charge transfer distance, amplifying the electronic configuration with adsorption strength for the oxygen intermediates, and synergism role of P dopant. Fe behaves as the active site and Co showed no detectable changes in valence state in their composite form. Henceforth is inert during the OER process. However, for the OER process, both Fe and Co must be active elements while performing the reaction. Hence, the group found that after the OER test, CoFeP was converted into the corresponding oxides and hydroxides on the catalyst surface and the newly formed compounds are in an amorphous face which reflected low overpotential and Tafel slope, indicating that the excellent electrocatalytic OER performance is due to Co, Fe, and P. All three entities participated in enhancing the OER activity. To further explore the ions' movement, electrochemical impedance spectroscopy (EIS) was conducted at different potentials to characterize the charge-transfer resistance (R_ct_) of samples (Figs. S3–S5). Illustrated the EIS curve, consisting of a semi-circular region at high frequency and a linear section at low frequency were obtained. The R_ct_ was related to the semicircle section that the smaller the diameter of the semicircle, the lower the R_ct_ and the more the charges can move. It was observed that CoFe-P exhibits an R_ct_ value of 1.832 Ω when compared to CoFe-S and CoFe (2.034 and 2.453 Ω). The lower value of R_ct_ in CoFe-P demonstrates the improvement in the callus nature of kinetics of the water-splitting to a higher degree, which was consistent with the LSV. Henceforth, the higher charge transfer coefficient corresponds to a faster charge transfer rate with low resistance was beneficial to improve the catalytic activity during the electrolysis process. Moreover, the phosphide sample is better than sulfide in the HER and OER process (Video S1), which can be reasoned to the lower electronegativity of P, and the ability to acquire the interstitial position between the atoms of the Co–Fe. due to its optimum size, therefore, it behaves as a site of positive ions and is rich in trapping positively charged species, such as H_2_ [16]. However, metals are electropositive and prefer radical-containing species to entangle and behave as active sites. Therefore, the electrocatalytic property of nanocomposite can be enhanced by the incorporation of P into bimetallic materials.

Further, to explore an extensive mechanism, CV curves at varied scan rates were recorded to obtain the C_dl_ of CoFe-P which is proportional to electrochemical active surface area (ECSA), which was observed under a non-faradic region of cyclic voltammogram (CVs) and double layer capacitance (C_dl_) was calculated by deriving a slope from the current vs scan rate curve showed in Fig. 5e, defined by an Eq. (10).

10 $${i}_{c}=v{C}_{dl}$$

The measured C_dl_ for CoFe-P is 82.1 mF, which is converted to ECSA by using Eq. 11. ECSA is directly proportional to the C_dl_ value. Thereby, the higher the C_dl_, the larger the ECSA, more the exposure of active sites on the surface of the catalysts.

11 $$ECSA= \frac{{C}_{dl}}{{C}_{s}}$$

12 $$RF= \frac{ECSA}{GSA}$$

where C_s_ is the specific capacitance, in this context the value of C_s_ is 0.040 mF/cm^2^ in 1 M KOH which is taken from the literature and previous reports [24]. Hence ECSA of 2000 cm^2^ was obtained for CoFe-P and a slightly lower value of ECSA was obtained for CoFe-S. Further, it was clearly distinguished by calculating the roughness factor (RF) [43], using Eq. 12 (GSA stands for the geometric surface area), indicating excellent gas bubble dissipation capability. Figure 5f delineates the ECSA and RF, CoFe-P illustrated a higher RF value (5847.6) than the CoFe-S and CoFe, 3883.1 and 1020.8, respectively. Therefore, CoFe-P assessed the larger density of the active sites on the surface and has the potential to dissipate a surplus amount of H_2_ and O_2_ gas in the water-splitting process. Hence considered the best candidate for electrocatalytic operation. Moreover, to understand the OER mechanism and active site, we also performed the DFT calculation similar to HER, except optimizing the OER intermediates. When compared to the CoFe and CoFe-S samples, the CoFe-P sample with Co-site as the main OER active site demonstrated the best catalytic candidacy Fig. 5g–h. The reasons for the good catalytic activity of CoFe-P can be attributed to factors similar to HER

**Fig. 6:**
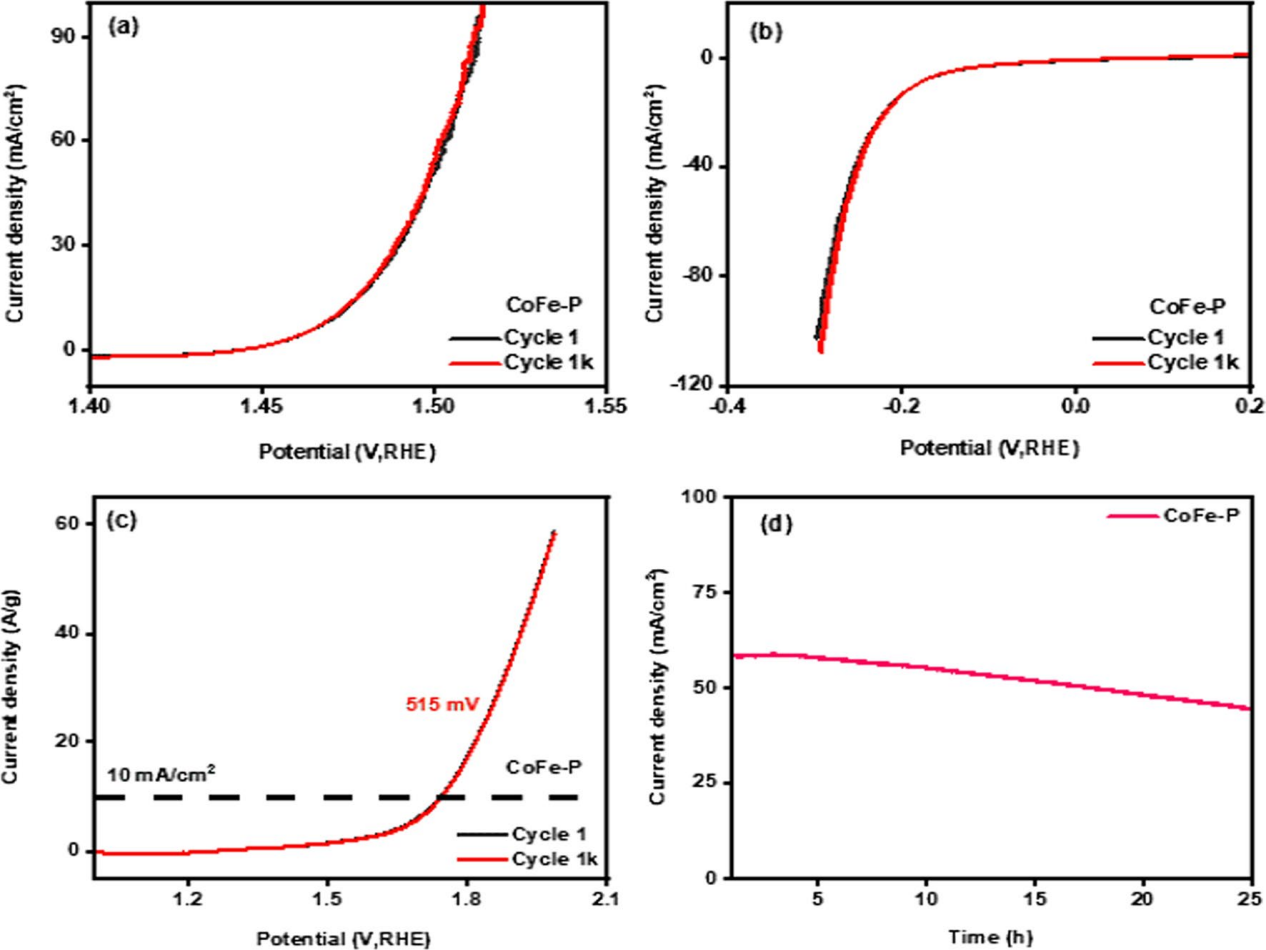
**a** and **b** 1 and 1 k OER and HER polarization curve, respectively, **c** LSV curve for electrolyzer and stability over 1000 cycles, **d** chronoamperometry curve for 24 h

All the prepared electrodes showed overlapping of the 1st polarization curve and the polarization curve after performing 1 k cycles. The polarization curve of CoFe-P for HER and OER are shown in Fig. 6a and b and are considered exceptionally durable electrocatalysts. Figure S6a–d demonstrated the polarization curve for CoFe and CoFe-S nanocomposites. Further, chronoamperometry confirmed the stability when the electrode was analyzed for 24 h and portrayed better performance without minimal deviation in current density. Figure S7a–c shows the CA curves of all the prepared nanocomposites. The variation in current density observed at the beginning might be due to the collection and release of bubbles produced by the splitting mechanism. Figure S7d depicts the CV curve for CoFe-P nanocomposite to better understand its stability during electrocatalyatic activity. The before and after CV curve overlap each other indicating the performance was very stable. Table S3 displayed a comparison between the electrocatalytic activity of as-synthesized samples with the other reported samples.

Moreover, the synthesized materials showed excellent behavior for HER and OER performances, due to their improved water-splitting nature, the best sample among all was tested for two electrode-electrolyzer testing for a real-life application (Video S2). This is the alternative and renewable method to produce H_2_ as fuel and O_2_ for medical purposes [49]. The system was assembled in 1 M basic electrolyte. The electrode was paired as a cathode and anode for Co–Fe–P. As shown in Fig. 6c, the obtained overpotential for the electrolyzer at 10 mA/cm^2^ was 515 mV. The voltage lag for the HER and OER processes evaluated in both the 3-electrode system and the 2-electrode-electrolyzer was noted to be 275 mV and 355 mV at 10 mA/cm^2^. The test was conducted for 1000 cycles and after the 1st and 1000th cycles of the polarization, the curve was quite matched with the 1000th cycle of the curve, indicating good durability. Furthermore, CA as shown in Fig. 6d measurement was performed, and the stability of the material was observed for 24 h, indicating high activity and the sustenance of a stable current density from 59 to 50 mA/cm^2^ with the production of H_2_ and O_2_ gases at the cathode and anode, respectively, hence highlighting its promise as a good candidate for low-cost clean energy production.

#### Supercapacitor studies

**Fig. 7:**
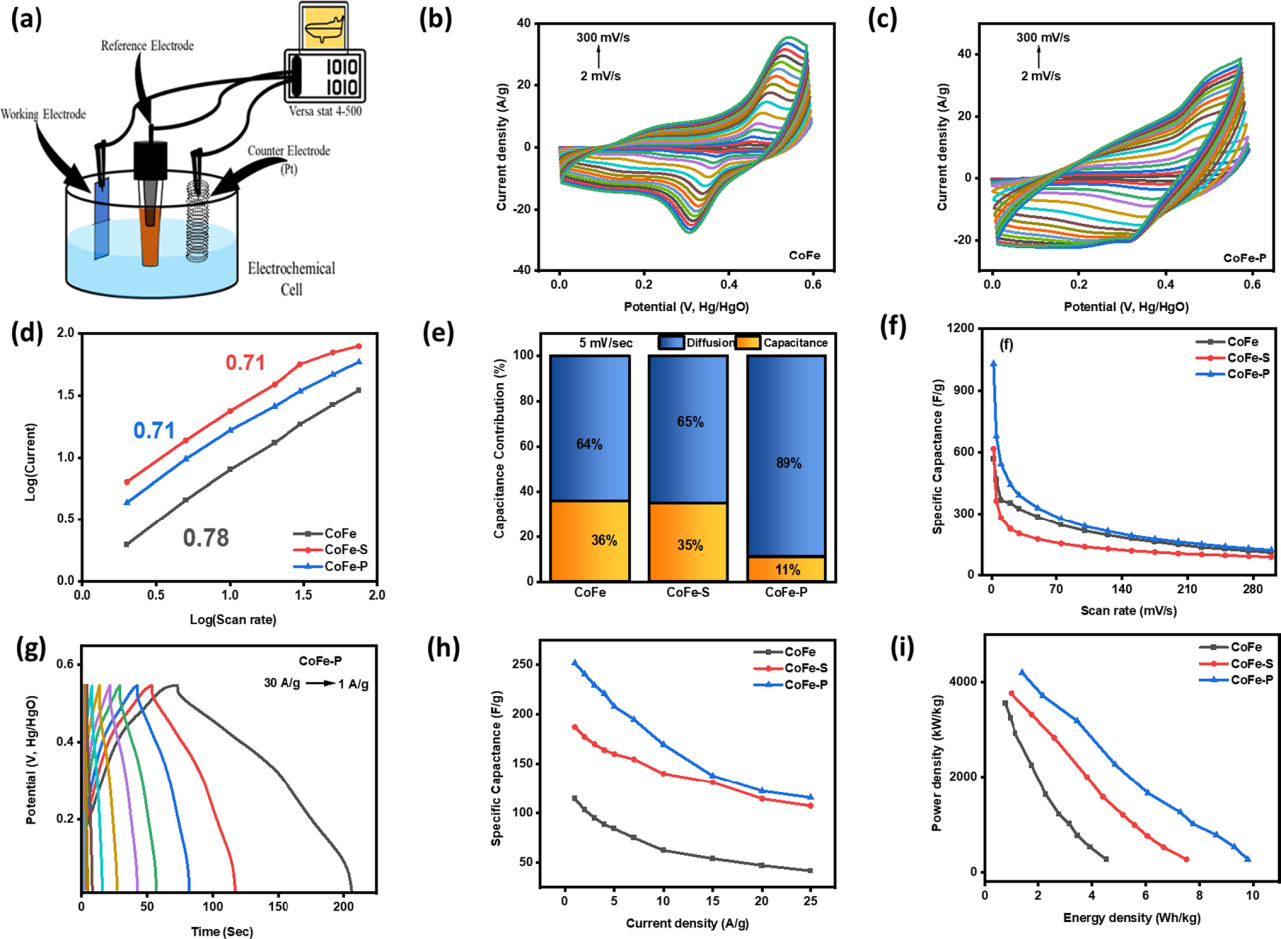
Electrochemical performance: **a** depicts the schematic three-electrode system. **b** and **c** CV scan curves from 2 to 300 mV/s of CoFe and CoFe-P. **d** log(current) *v/s* log(scan rate), **e** shows the diffusion and capacitance contribution at 5 mV/s, and **f** depicts the C_sp_ (F/g) *v/s* scan rate (mV/s) curve. **g** charge–discharge curves of CoFe-P nanocomposites at various current density ranging from 30–1 A/g. **h** Shows the variation of C_sp_ (F/g) with current density (A/g), **i** shows the variation of power and energy density (Ragone Plot)

The SC performance of as-prepared nanocomposites was measured at room temperature in an operating voltage window of 0–0.6 V. The schematic diagram of the working of the three-electrode system is shown in Fig. 7a. The cyclic voltammetry (CV), galvanostatic charge–discharge (GCD), electrochemical impedance spectroscopy (EIS), and stability were employed to confirm the charge storage phenomenon types along with the amount of C_sp_. The CV curves indicate the type of charge storage mechanism, either a pseudocapacitance or electric double-layer capacitance (EDLC). Pseudocapacitance refers to reversible redox reactions showing the intercalation and electro-sorption on the surface of electrodes. Moreover, EDLC forms a double layer on the surface of the electrodes and electrolyte interface. Figure 7b and c represents the typical CV curves for CoFe, CoFe-S (Fig. S8), and CoFe-P nanocomposites. The CoFe electrodes exhibit redox peaks showing the reversible redox nature. The redox behavior is supported by the following reactions during the CV, as shown in Eqs. (13)–(15) [50, 51].

13 $${\text{CoFe}}_{{2}} {\text{O}}_{{4}} + {\text{OH}}^{ - } \leftrightarrow {\text{CoFe}}_{{2}} {\text{O}}_{{{4} - {\text{z}}}} {\text{OH}}_{{\text{z}}} + {\text{H}}_{{2}} {\text{O}} + {\text{e}}^{ - }$$

14 $${\text{Co}}_{{8}} {\text{FeS}}_{{8}} + {\text{OH}}^{ - } \leftrightarrow {\text{Co}}_{{8}} {\text{FeS}}_{{{8} - {\text{x}}}} {\text{OH}}_{{\text{x}}} + {\text{H}}_{{2}} {\text{O}} + {\text{e}}^{ - }$$

15 $${\text{Co}}_{{1}} {\text{Fe}}_{{0.{1}}} {\text{P}} + {\text{OH}}^{ - } \leftrightarrow {\text{Co}}_{{1}} {\text{Fe}}_{{0.{1}}} {\text{P}}_{{{1} - {\text{y}}}} {\text{OH}}_{{\text{y}}} + {\text{H}}_{{2}} {\text{O}} + {\text{e}}^{ - }$$

The reversible redox behavior is supported through anodic peaks between 0.45 and 0.6 V, while the cathodic peak lies between 0.2 and 04 V showing a transition between Co^3+^ ↔ Co^2+^, Fe^3+^ ↔ Fe^2+^. The shift in peaks is due to the varied scan rate. Furthermore, minimal anodic and cathodic peaks were obtained during the CoFe-S and CoFe-P nanocomposite showing the combination of both pseudo and EDLC behavior. The peak-to-peak separation (ΔE_p_) varies inversely with the charge transfer rate (K_s_). The ΔE_p_ at 100 mV/sec scan rate for CoFe, CoFe-S, and CoFe-P nanocomposite are as follows 0.191, 0.163 and 0.112 V confirming the highest K_s_ for phosphorized nanocomposite [52]. The K_s_ must be the reason for better ionic mobility between the electrode/electrolyte interface compared to other prepared nanocomposites.

The CoFe-P exhibits the highest current density along with the highest surface area under the CV curve, which further implies that more charge can be stored on the surface of the electrode. Moreover, the charge storage mechanism is confirmed using the given Eq. (16).

16 $$i = av^{b}$$

where *i* represents the peak current, *v* is the scan rate where *b* is the slope of the plot log (scan rate), *v/s* log (current), and *a* is the intercept on the Y-axis. The value of *b* = 0.5 indicates the diffusion-controlled faradaic reaction. The purely capacitive mechanism was demonstrated when *b* = 1. Here, Fig. 7d shows the log(current), *v/s* log (scan rate) curve where, CoFe, CoFe-S and CoFe-P nanocomposite exhibit slopes 0.78, 0.71 and 0.71 demonstrating the hybrid type of behavior. In addition, the type of capacitance contribution at various scan rates can be calculated based on the given Eq. (17).

17 $$i = {\text{k}}_{{1}} v + {\text{k}}_{{2}} v^{{{1}/{2}}}$$

where k_1_*v* depicts the capacitive nature and k_2_*v*^*1/2*^ indicates the diffusion effect. Figure 7e shows the diffusion and capacitance contribution at 5 mV/sec. The CoFe nanocomposite shows a 64% diffusive and 36% capacitive nature, whereas the CoFe-S and CoFe-P exhibit 65 and 89% diffusion dedicating better penetrating properties. Therefore, the improvement in electrochemical storage properties has been observed while going from CoFe to CoFe-S to CoFe-P. The above fact is supported in Fig. 7f, where the C_sp_ *v/s* scan rate curve shows the highest C_sp_ of 1042 F/g at 2 mV/sec for the CoFe-P nanocomposite.

GCD curves have been employed at various current densities (1–30 A/g) to understand the quantitative charge storage mechanism, where Fig. 7g, and (Fig. S9a–b) shows all the GCD curves. CoFe (Fig. S9a) shows the asymmetric triangular profile manifested in fast charge and slow discharge rate. CoFe-S (Fig. S9b) demonstrates the proper symmetric triangular profile indicating the well-balanced charge storage ability at higher and lower current densities. The discharge time for CoFe-P (Fig. 7g) is most elevated, showing the asymmetric triangular shape [53]. The highest C_sp_ for CoFe-P is 252 F/g at 1 A/g, which is more than twice as of CoFe nanocomposite. The C_sp_ is calculated based on the given Eq. (18), where *i* refers to the current, Δt indicates the discharge time, m represents the actual mass loading, and *ΔV* stands for the difference in applied voltage.

18 $${C}_{sp}=\frac{i\Delta t}{m\Delta V}$$

The charge storage property is supported based on FE-SEM images where CoFe shows an irregular globular structure. This results in low C_sp_ for CoFe nanocomposite. The above fact is supported by confirming the amorphous CoFe nanocomposite depicted through XRD. This leads CoFe towards fewer grain boundaries which further provides lesser active sites for OH^−^ ions to attack and release electrons.

Moreover, the CoFe-S nanocomposite shows a regular and more extensive globular morphology with improved crystallinity. The improvement in crystallinity provides more grain boundaries, further improving a more significant number of active sites for OH^−^ ions. Therefore, more electrons will be generated during the reaction, enhancing the C_sp_ [54]. However, the change in morphology has been observed while shifting towards CoFe-P nanocomposite. The globular molecules seem to be hollow spherical 3D globules. This increases the surface area of the nanocomposite, which is supported by the rise in intensity peaks of CoFe-P XRD along with higher crystalline nature. The plots C_sp_ *vs* current density and power *vs* energy density are shown in Fig. 7h and i, respectively.

**Fig. 8:**
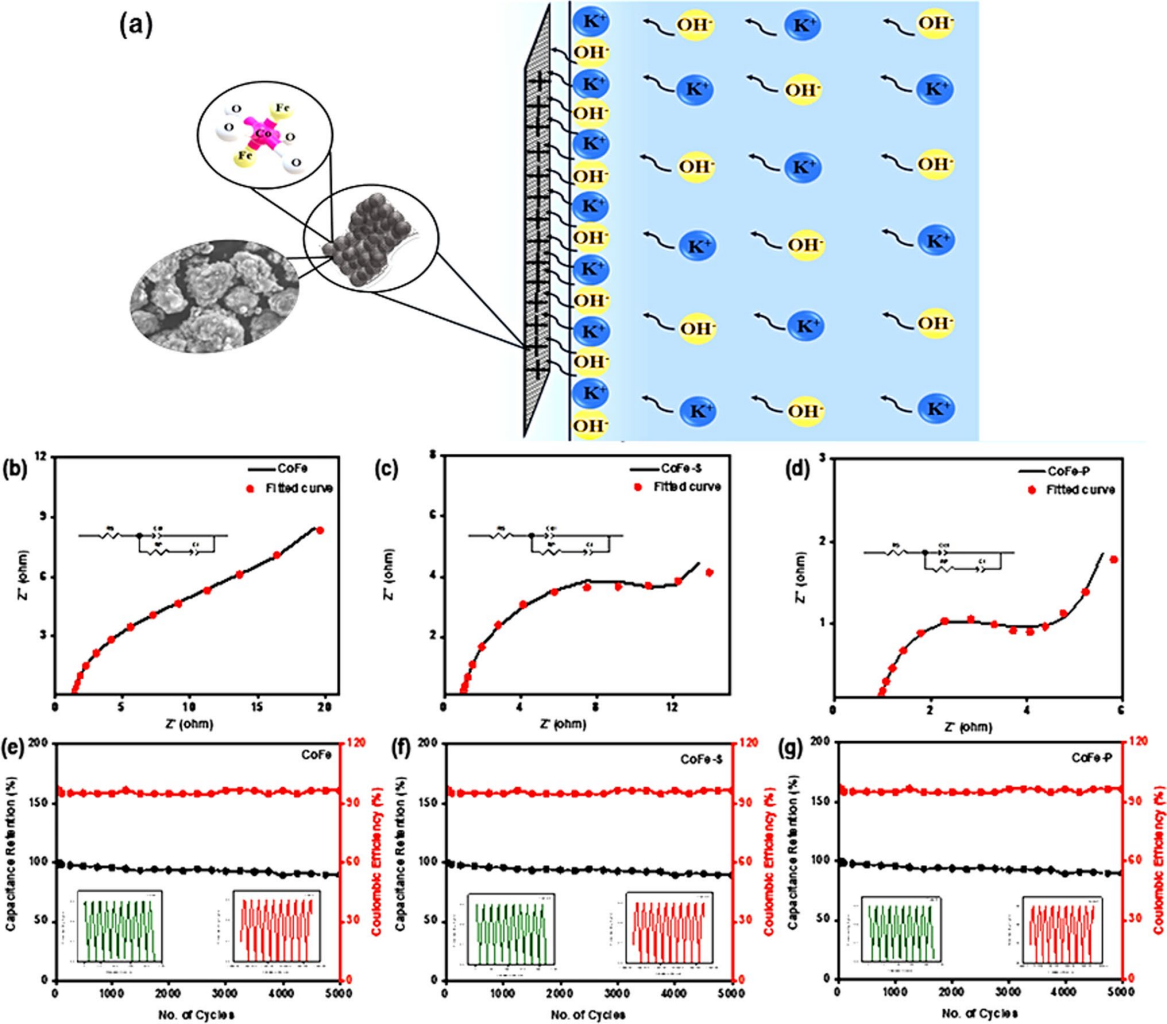
**a** Shows the ionic adsorption and desorption mechanism on the surface of the electrode. **b**–**d** Shows the EIS curve with the most fitted circuit, and **d**–**f** depicted the stability of all the as-synthesized nanocomposites

Furthermore, the electrochemical properties of nanocomposites were conducted using EIS measurements, where Nyquist plots (Fig. S10) were summarized along with the best-fitted circuit. The perfect semicircle in the Nyquist plot indicates the ideal capacitive behavior. In contrast, a semicircle with a vertical line indicates non-ideal capacitive behavior. The equivalent series resistance (ESR) is the combination of the ionic resistance of the electrolyte and the interfacial resistance at the surface of the active material. The intercept determines ESR at the real axis. The semicircle in the high-frequency region corresponds to charge transfer resistance (R_ct_) [55]. Z is the impedance of electrolyte ions calculated from the semicircle in the medium frequency range. The vertical line is caused by the diffusion resistance of electrolyte ions at a low-frequency range where the electrode deviates from the ideal capacitor behavior. Figure 8a shows the ionic adsorption and desorption mechanism on the surface of the electrode. The ESR value calculated from the graph and fitting the circuit match closely to each other. CoFe, CoFe-S, and CoFe-P nanocomposites exhibit 1.37, 0.95, and 0.91 Ω, ESR implying more ion–electron mobility, which further allows more charge to hold up on the surface. The addition of CPE (C_dl_), as shown in the circuit in Fig. 8b–d, depicts the non-ideal behavior of supercapacitor electrodes supporting the hybrid capacitive nature and stability is attributed in Fig. 8e–g [56]. Moreover, the details provided in Table S4 supported the above results strongly and confirmed the smooth charge transfer and mass transfer process for the CoFe-P nanocomposite.

The practical application of the device is stipulated by plotting the power density *v/s* energy density curve shown in Fig. 7i, including its calculative Eqs. (19)–(20).

19 $$E=\frac{1}{2}C\Delta {V}^{2}$$

20 $$P=\frac{E}{\Delta t}$$

The CoFe-P shows a 9.98 W–h/kg energy density and power density of 4137 W/kg. In addition, the stability test was investigated at 10 A/g up to 5000 cycles maintaining excellent cyclic performance with 97.2% capacitance retention, as shown in Fig. 8g. Table S5 shows the comparison between our sample and some other work. Noticeably, CoFe-P exhibits better cycle lives.

## Conclusions

Fast and broadly applicable hydrothermal methods were used to produce nanocomposites. The XRD pattern confirms their crystal structure which converts to hollow 3D-spherical globules from irregular globular morphology documented through SEM images which provides more active sites to the electrolyte for attacking and storing more charges. Moreover, the introduction of sulfur and phosphorus enhances the performance significantly. All prepared nanocomposites are subjected to electrocatalytic and SC studies where CoFe-P shows better performance with the lowest possible OER overpotential 240 mV, supported by the highest TOF value of 19.3/s showing better kinetics along with low ESR and charge transfer resistance with high discharge time for SC application. The CoFe-P offers 97.2% retention in capacitance, indicating its high stability. Through this research, excellent electrode material for electrocatalysts and SC application will help scientists develop futuristic electrode materials by modifying their morphological and intrinsic properties for application in electrolyzers and SCs.

## Supplementary Information

**Additional file 1: Figure S1(a-k)** The (a, d, and g)show the XPS spectra of O^1^s, Co 2p, and Fe 2p of as-synthesized CoFe, whereas (b,e, and h) follow the XPS spectra of O^1^s, Co 2p, and Fe 2p of CoFe-S and (c, f, and i) shows the XPS spectra of O^1^s, Co 2p, and Fe 2p of CoFe-P. (j and k) shows the XPS spectra of S in CoFe-S and P in CoFe-P. **Figure S2** (a–c) The XPS Survey for (a) CoFe, (b) CoFe-S, and (c) CoFe-P Sample. **Figure S3** EIS of CoFe sample at the with different applied potentials. **Figure S4** EIS of CoFe-S sample at the different applied potentials. **Figure S5** EIS of CoFe-P sample at the different applied potentials. **Figure S6** (a–d) Shows the polarization curve of OER and HER for CoFe, and CoFe-S nanocomposite. **Figure S7** (a–c) Shows the CA curves for all the as-prepared CoFe, CoFe-S, and CoFe-P-nanocomposite. (d) shows the CV curves before and after CA testing of CoFe-P. **Figure S8** CV scan curves from 2 to 300 mV/s for CoFe-S. **Figure S9** (a, b) The charge-discharge curves of CoFe, and CoFe-S at various current density ranging from 30-1 A/g. **Figure S10** Shows the Nyquist plots of all the as-prepared CoFe, CoFe-S, and CoFe-P-nanocomposite. **Video S1** Shows the three-electrode system for HER/OER. **Video S2** Shows the electrolyzer testing. **Table S1** Comparison of the synthesized samples for HER electrocatalytic activity on different parameters. **Table S2** Comparison of the synthesized samples for OER electrocatalytic activity on different parameters. **Table S3** Comparison of the previously reported electrocatalysts for HER and OER. **Table S4** All the calculated values for ESR and C_dl_ are depicted below. **Table S5** Comparison of the previously reported capacitance values.

## Data Availability

The datasets generated during and/or analysed during the current study are available from the corresponding author on reasonable request.
