# Supplementary material for: Bimetallic Co–Fe sulfide and phosphide as efficient electrode materials for overall water splitting and supercapacitor
Source: Discov Nano. 2023 Apr 4;18(1):59. doi: 10.1186/s11671-023-03837-1 (PMC10409961; doi:10.1186/s11671-023-03837-1)
Supplement: Supplementary file 1 — Additional file 1: Figure S1(a-k) The (a, d, and g)show the XPS spectra of O1s, Co 2p, and Fe 2p of as-synthesized CoFe, whereas (b,e, and h) follow the XPS spectra of O1s, Co 2p, and Fe 2p of CoFe-S and (c, f, and i) shows the XPS spectra of O1s, Co 2p, and Fe 2p of CoFe-P. (j and k) shows the XPS spectra of S in CoFe-S and P in CoFe-P. Figure S2 (a–c) The XPS Survey for (a) CoFe, (b) CoFe-S, and (c) CoFe-P Sample. Figure S3 EIS of CoFe sample at the with different applied potentials. Figure S4 EIS of CoFe-S sample at the different applied potentials. Figure S5 EIS of CoFe-P sample at the different applied potentials. Figure S6 (a–d) Shows the polarization curve of OER and HER for CoFe, and CoFe-S nanocomposite. Figure S7 (a–c) Shows the CA curves for all the as-prepared CoFe, CoFe-S, and CoFe-P-nanocomposite. (d) shows the CV curves before and after CA testing of CoFe-P. Figure S8 CV scan curves from 2 to 300 mV/s for CoFe-S. Figure S9 (a, b) The charge-discharge curves of CoFe, and CoFe-S at various current density ranging from 30-1 A/g. Figure S10 Shows the Nyquist plots of all the as-prepared CoFe, CoFe-S, and CoFe-P-nanocomposite. Video S1 Shows the three-electrode system for HER/OER. Video S2 Shows the electrolyzer testing. Table S1 Comparison of the synthesized samples for HER electrocatalytic activity on different parameters. Table S2 Comparison of the synthesized samples for OER electrocatalytic activity on different parameters. Table S3 Comparison of the previously reported electrocatalysts for HER and OER. Table S4 All the calculated values for ESR and Cdl are depicted below. Table S5 Comparison of the previously reported capacitance values. [file 11671_2023_3837_MOESM1_ESM.docx]

**Bimetallic Co-Fe Sulfide and Phosphide as Efficient Electrode Materials for Overall Water Splitting and Supercapacitor**

**Shiva Bhardwaj^1,2^, Rishabh Srivastava^1,2^, Teddy Mageto^1,2^, Anuj Kumar^5*^, Mahesh Chaudhari^2,6^, Jolaikha Sultana^3^, Sanjay R. Mishra^3^, Felio Perez^4^ and Ram Gupta^2,6*^**

^1^Department of Physics, Pittsburg State University, Pittsburg, Kansas 66762, USA

^2^National Institute of Material Advancement, Pittsburg State University, Pittsburg, Kansas 66762, USA

^3^Department of Physics and Materials Science, The University of Memphis, Memphis, TN 38152, USA

^4^Integrated Microscopy Center, The University of Memphis, Memphis, TN 38152, USA

^5^Nano-Technology Research Laboratory, Department of Chemistry, GLA University, Mathura, Uttar Pradesh 281406, India

^6^Department of Chemistry, Pittsburg State University, Pittsburg, Kansas 66762, USA


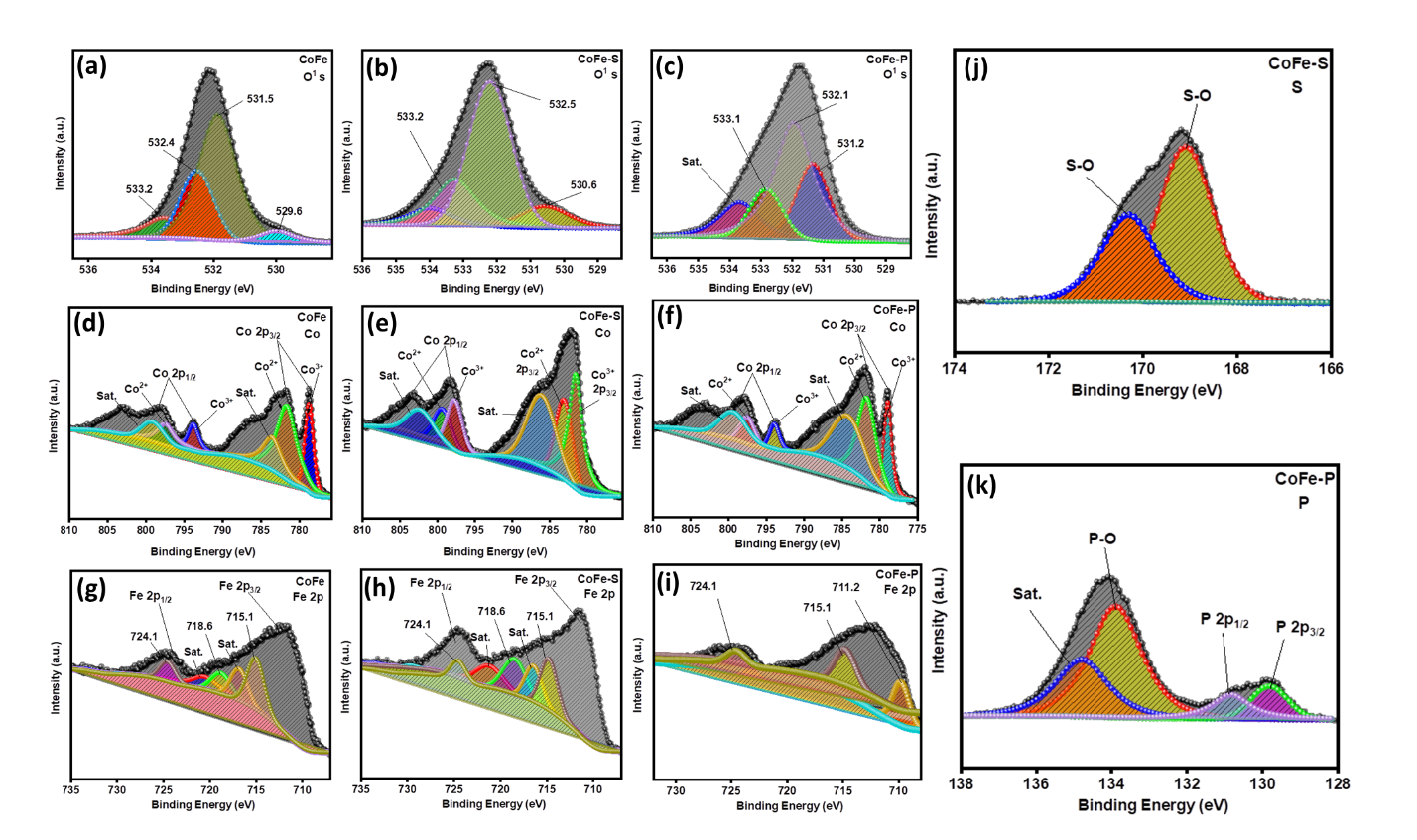


**Figure S1(a-k):** The **(a, d, and g)** show the XPS spectra of O^1^s, Co 2p, and Fe 2p of as-synthesized CoFe, whereas **(b,e, and h)** follow the XPS spectra of O^1^s, Co 2p, and Fe 2p of CoFe-S and **(c, f, and i)** shows the XPS spectra of O^1^s, Co 2p, and Fe 2p of CoFe-P. **(j and k)** shows the XPS spectra of **S** in CoFe-S and **P** in CoFe-P.

.


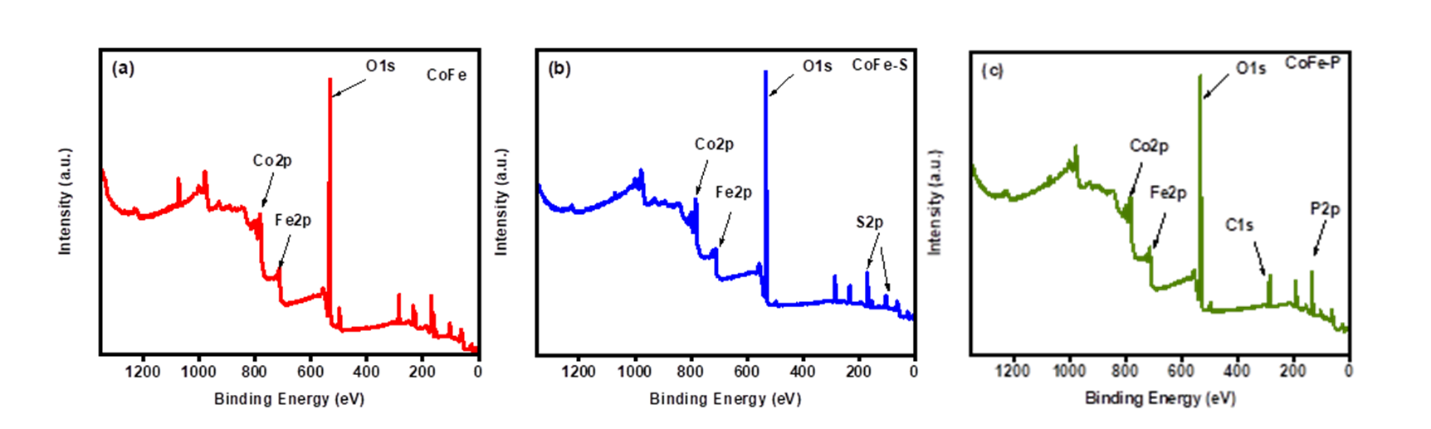


**Figure S2(a-c):** The XPS Survey for (a) CoFe, (b) CoFe-S, and (c) CoFe-P Sample.

**Figure S3:** EIS of CoFe sample at the with different applied potentials.

**Figure S4:** EIS of CoFe-S sample at the different applied potentials.

**Figure S5:** EIS of CoFe-P sample at the different applied potentials.

**Figure S6:** (a-d) Shows the polarization curve of OER and HER for CoFe, and CoFe-S nanocomposite.

**Figure S7:** (a-c) Shows the CA curves for all the as-prepared CoFe, CoFe-S, and CoFe-P-nanocomposite. (d) shows the CV curves before and after CA testing of CoFe-P.

**Figure S8:** CV scan curves from 2 to 300 mV/s for CoFe-S.

**Figure S9:** (a-b) The charge-discharge curves of CoFe, and CoFe-S at various current density ranging from 30-1 A/g.

**Figure S10:** Shows the Nyquist plots of all the as-prepared CoFe, CoFe-S, and CoFe-P-nanocomposite.


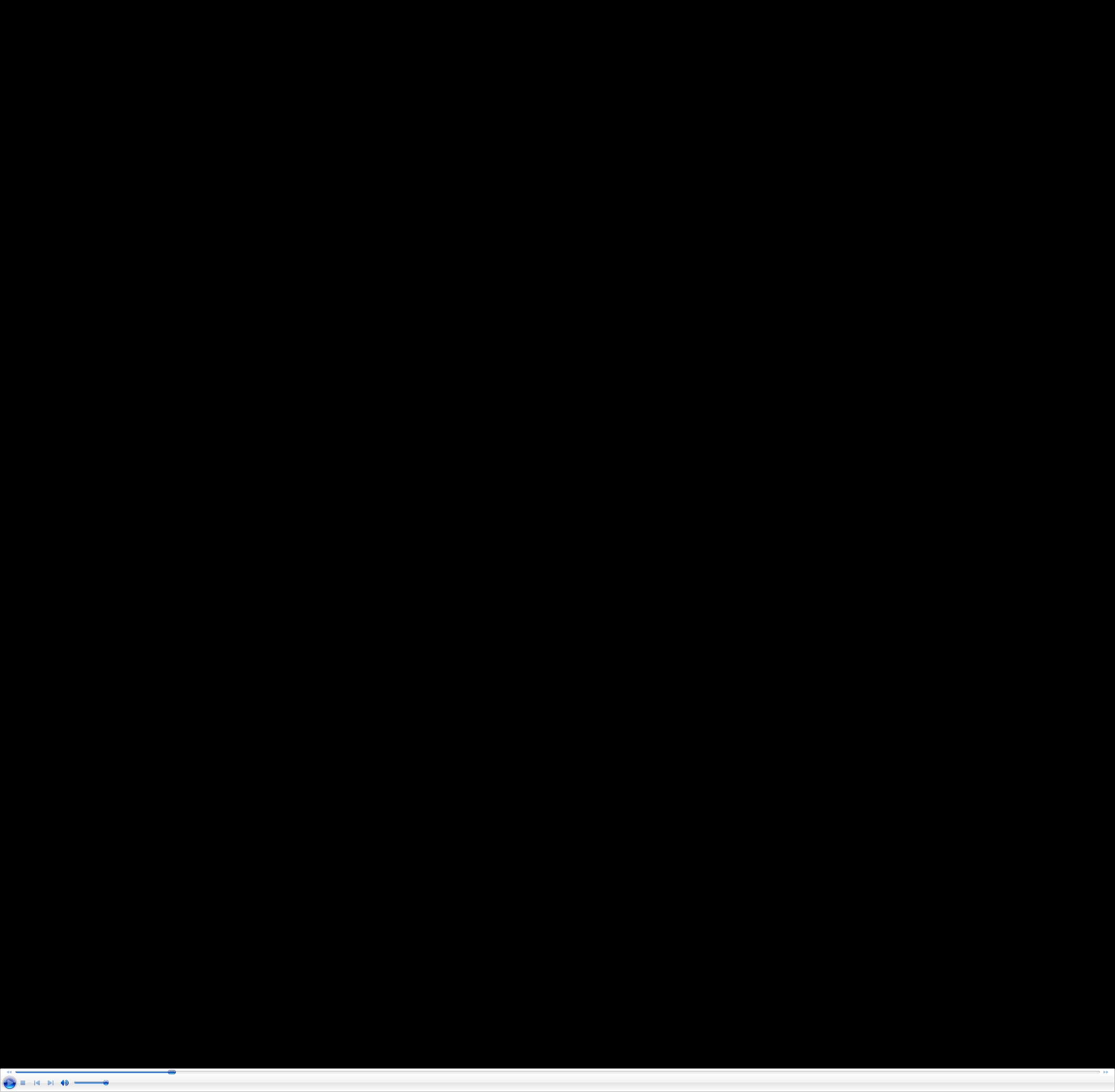


**Video S1:** Shows the three-electrode system for HER/OER.


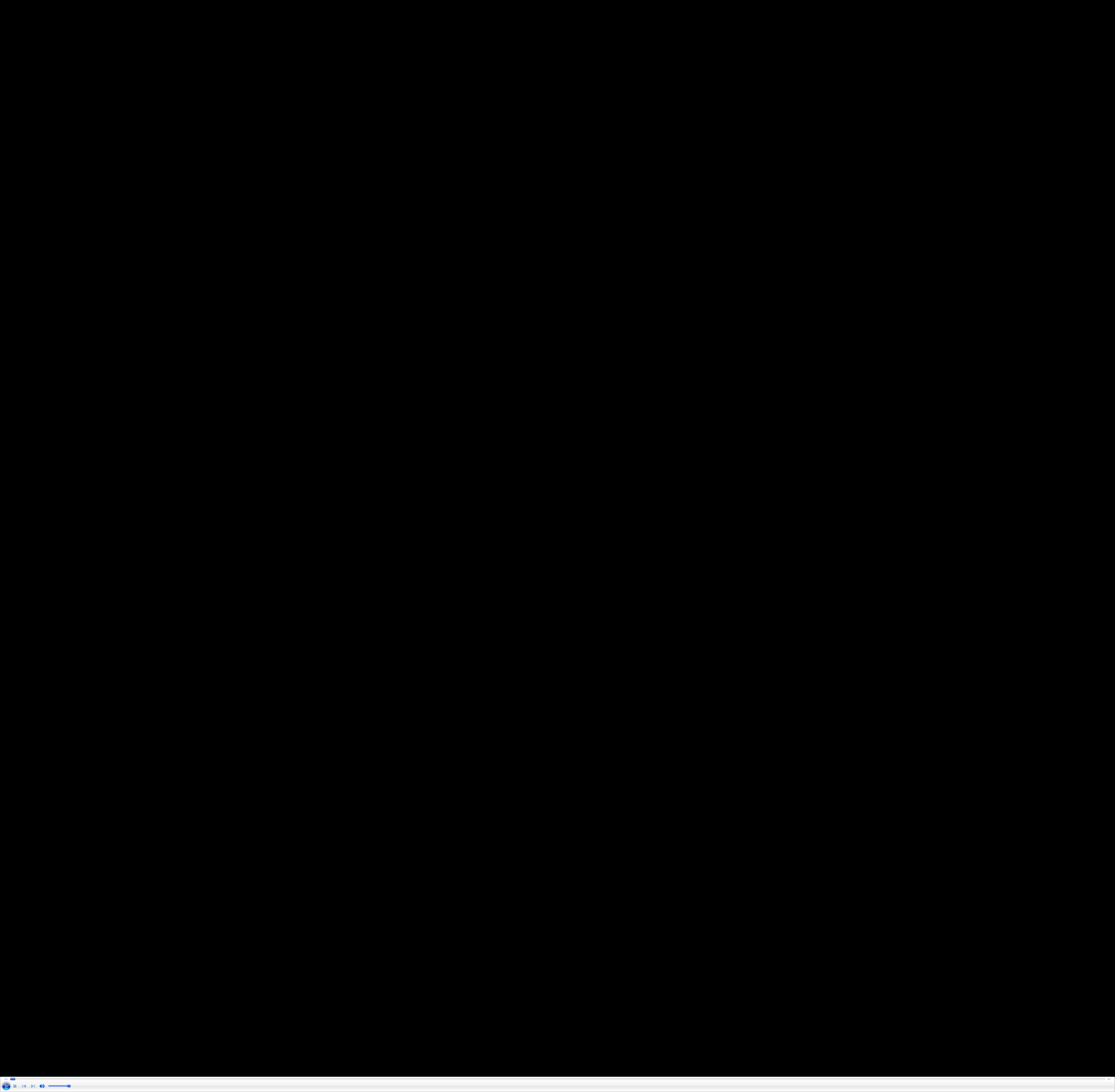


**Video S2:** Shows the electrolyzer testing.

**Table S1.** Comparison of the synthesized samples for HER electrocatalytic activity on different parameters.

| **Sample** | **Overpotential (mV)**  **@ 10 mA/cm^2^** | **Tafel slope**  **(mV/dec)** | **TOF (s^-1^)**  **@ 250 mV** | **MA (A/g)**  **@ 250 mV** |
| --- | --- | --- | --- | --- |
| CoFe | 208 | 132 | 0.864 | 2.3208 |
| CoFe-S | 229 | 118 | 4.221 | 3.0081 |
| CoFe-P | 186 | 113 | 5.411 | 4.2390 |

**Table S2.** Comparison of the synthesized samples for OER electrocatalytic activity on different parameters.

| **Sample** | **Overpotential (mV)**  **@ 10 mA/cm^2^** | **Tafel slope**  **(mV/dec)** | **TOF**  **(s^-1^)**  **@ 300 mV** | **MA (A/g)**  **@ 300 mV** | **C_dl_**  **(mF)** | **ECSA**  **(cm^2^)** | **RF** |
| --- | --- | --- | --- | --- | --- | --- | --- |
| CoFe | 263 | 43 | 1.075 | 10.7522 | 19.8 | 495 | 1020.82 |
| CoFe-S | 246 | 59 | 11.954 | 14.4297 | 80.1 | 2002 | 3883.07 |
| CoFe-P | 240 | 36 | 19.300 | 17.9558 | 82.1 | 2052 | 5847.57 |

**Table S3.** Comparison of the previously reported electrocatalysts for HER and OER.

|  | HER |  | OER |  |  |
| --- | --- | --- | --- | --- | --- |
| Sample | **Overpotential (mV)**  **@10 mA/cm^2^** | **Tafel slope**  **(mV/dec)** | **Overpotential (mV)**  **@10 mA/cm^2^** | **Tafel slope**  **(mV/dec)** | **References** |
| Ni-CO-P HNBs | 107 | 46 | 270 | 76 | [1] |
| CTGU-10c2 | 240 | 58 | - | - | [2] |
| FeCoP UNSAs | 188@100 mA/cm^2^ | 76 | 330@100 mA/cm^2^ | 63 | [3] |
| Co_x_Fe_1-x_ WO4 | - | - | 327 | 53 | [4] |
| NF@NC-CoFe_2_O_4_/C NRAs | - | - | 240 | 45 | [5] |
| CoFe-LDH | 194 | 127 | - | - | [6] |
| CoFe_2_O_4_ ED@NF | - | - | 270 | 31 | [7] |
| CF-CFO/NC | 26 | 113 | 243 | 78 | [8] |
| NiCo-UMOFNs | - | - | 250 | 65 | [9] |
| Fe_1_Co_3_O_x_@C-800 | - | - | 270 | 40 | [10] |
| Fe_1.89_Mo_4.11_O_7_/Mo_2_ | 197 | 79 | - | - | [11] |
| Fe_3_C-Co/NC | 238 | - | 371 | - | [12] |
| PPy/FeTCPP/Co | 270 | 155 | 340 | 61 | [13] |
| Co/N-C | 212 | 75 | 400 | 60 | [14] |
| CoFe-LDH/NF | 320 | 127 | 240 | 65 | [15] |
| CoFe-S | **208** | **118** | **246** | **43** | **This work** |
| CoFe-P | **186** | **113** | **240** | **36** | **This work** |

**Table S4.** All the calculated values for ESR and C_dl_ are depicted below.

| **Sample** | **R_s_ (Ω)** | **R_ct_ (Ω)** | **C_dl_ (F/g)** |
| --- | --- | --- | --- |
| CoFe | 1.374 | 5.321 | 0.8 |
| CoFe-S | 0.95 | 4.221 | 1.8 |
| CoFe-P | 0.91 | 2.832 | 2.4 |

**Table S5:** Comparison of the previously reported capacitance values.

| **Sample** | **Specific Capacitance (F/g @1 A/g)** | **Energy Density (W-h/kg)** | **Power Density (W/kg)** | **References** |
| --- | --- | --- | --- | --- |
| rGO/CoFe_2_O_4_ | 164 | 22.8 | 410 | [16] |
| CoFe_2_O_4_ | 15 | 1.2 | 108 | [17] |
| CoFeS_2_ | 169 | 3.2 | 796 | [18] |
| NiCoFeP | 136 | 4.8 | 811 | [19] |
| CoHCFe | 250 | 34.2 | 2500 | [20] |
| CoFeSi | 96 | 6.5 | 68.8 | [21] |
| h-CoFe-MoS_x_ | 85.4 | 1.9 | 322 | [22] |
| CoFe_2_O_4_ | 195 | 12.14 | 643 | [23] |
| Co_2_P-nanoflower | 416 | 8.8 | 6000 | [24] |
| Co_2_P-nanorod | 284 | 6.2 | 4362 | [24] |
| **CoFe** | **120** | **4.3** | **3752** | **This work** |
| **CoFe-S** | **183** | **7.9** | **3892** | **This work** |
| **CoFe-P** | **252** | **9.98** | **4137** | **This work** |

**References**

[1] E. Hu, Y. Feng, J. Nai, D. Zhao, Y. Hu, X.W. (David) Lou, Construction of hierarchical Ni–Co–P hollow nanobricks with oriented nanosheets for efficient overall water splitting, Energy Environ. Sci. 11 (2018) 872–880.

[2] W. Zhou, D.-D. Huang, Y.-P. Wu, J. Zhao, T. Wu, J. Zhang, D.-S. Li, C. Sun, P. Feng, X. Bu, Stable Hierarchical Bimetal-Organic Nanostructures as HighPerformance Electrocatalysts for the Oxygen Evolution Reaction., Angew. Chem. Int. Ed. Engl. 58 (2019) 4227–4231.

[3] Y. Liu, S. Liu, Y. Wang, Q. Zhang, L. Gu, S. Zhao, D. Xu, Y. Li, J. Bao, Z. Dai, Ru Modulation Effects in the Synthesis of Unique Rod-like Ni@Ni(2)P-Ru Heterostructures and Their Remarkable Electrocatalytic Hydrogen Evolution Performance., J. Am. Chem. Soc. 140 (2018) 2731–2734.

[4] J. Ma, S. Zheng, F. Zhou, Y. Zhu, P. Das, R. Huang, L. Zhang, X. Wang, H. Wang, Y. Cui, Z.-S. Wu, All 3D printing lithium metal batteries with hierarchically and conductively porous skeleton for ultrahigh areal energy density, Energy Storage Mater. (2022).

[5] T. Li, Y. Lv, J. Su, Y. Wang, Q. Yang, Y. Zhang, J. Zhou, L. Xu, D. Sun, Y. Tang, Anchoring CoFe2O4 Nanoparticles on N-Doped Carbon Nanofibers for High-Performance Oxygen Evolution Reaction, Adv. Sci. 4 (2017) 1700226.

[6] S. Liu, J. Zhu, M. Sun, Z. Ma, K. Hu, T. Nakajima, X. Liu, P. Schmuki, L. Wang, Promoting the hydrogen evolution reaction through oxygen vacancies and phase transformation engineering on layered double hydroxide nanosheets, J. Mater. Chem. A. 8 (2020) 2490–2497.

[7] C. Zhang, S. Bhoyate, C. Zhao, P.K. Kahol, N. Kostoglou, C. Mitterer, S.J. Hinder, M.A. Baker, G. Constantinides, K. Polychronopoulou, C. Rebholz, R.K. Gupta, Electrodeposited Nanostructured CoFe2O4 for Overall Water Splitting and Supercapacitor Applications, Catalysts. 9 (2019) 176.

[8] H. Zhuang, Y. Xie, H. Tan, Y. Deng, Y. Li, G. Chen, CoFex-CoFe2O4/N-doped carbon nanocomposite derived from in situ pyrolysis of a single source precursor as a superior bifunctional electrocatalyst for water splitting, Electrochim. Acta. 262 (2018) 18–26.

[9] S. Zhao, Y. Wang, J. Dong, C.-T. He, H. Yin, P. An, K. Zhao, X. Zhang, C. Gao, L. Zhang, J. Lv, J. Wang, J. Zhang, A.M. Khattak, N.A. Khan, Z. Wei, J. Zhang, S. Liu, H. Zhao, Z. Tang, Ultrathin metal–organic framework nanosheets for electrocatalytic oxygen evolution, Nat. Energy. 1 (2016) 16184.

[10] X. Bai, Q. Wang, J. Guan, Bimetallic Iron-Cobalt Nanoparticles Coated with Amorphous Carbon for Oxygen Evolution, ACS Appl. Nano Mater. 4 (2021) 12663–12671.

[11] Z. Hao, S. Yang, J. Niu, Z. Fang, L. Liu, Q. Dong, S. Song, Y. Zhao, A bimetallic oxide Fe1.89Mo4.11O7 electrocatalyst with highly efficient hydrogen evolution reaction activity in alkaline and acidic media, Chem. Sci. 9 (2018) 5640–5645.

[12] C.C. Yang, S.F. Zai, Y.T. Zhou, L. Du, Q. Jiang, Fe3C-Co Nanoparticles Encapsulated in a Hierarchical Structure of N-Doped Carbon as a Multifunctional Electrocatalyst for ORR, OER, and HER, Adv. Funct. Mater. 29 (2019) 1–12.

[13] J. Yang, X. Wang, B. Li, L. Ma, L. Shi, Y. Xiong, H. Xu, Novel Iron/Cobalt-Containing Polypyrrole Hydrogel-Derived Trifunctional Electrocatalyst for Self-Powered Overall Water Splitting, Adv. Funct. Mater. 27 (2017) 1606497.

[14] Z. Pei, Z. Tang, Z. Liu, Y. Huang, Y. Wang, H. Li, Q. Xue, M. Zhu, D. Tang, C. Zhi, Construction of a hierarchical 3D Co/N-carbon electrocatalyst for efficient oxygen reduction and overall water splitting, J. Mater. Chem. A. 6 (2018) 489–497.

[15] R. Yang, Y. Zhou, Y. Xing, D. Li, D. Jiang, M. Chen, W. Shi, S. Yuan, Synergistic coupling of CoFe-LDH arrays with NiFe-LDH nanosheet for highly efficient overall water splitting in alkaline media, Appl. Catal. B Environ. 253 (2019) 131–139.

[16] S. Ishaq, M. Moussa, F. Kanwal, R. Ayub, T.N. Van, U. Azhar, D. Losic, One step strategy for reduced graphene oxide/cobalt-iron oxide/polypyrrole nanocomposite preparation for high performance supercapacitor electrodes, Electrochim. Acta. 427 (2022) 140883.

[17] S.J. Pawar, S.M. Patil, M. Chithra, S.C. Sahoo, P.B. Patil, Cobalt ferrite nanoparticles for supercapacitor application, AIP Conf. Proc. 2265 (2020).

[18] M. Karuppannan, Y. Kim, D. Lee, Y.-E. Sung, O.J. Kwon, Ball mill assisted synthesis of cobalt–iron sulfide/N-doped carbon for high performance asymmetric supercapacitors, J. Appl. Electrochem. 50 (2020) 1119–1128.

[19] G. Qu, P. Sun, G. Xiang, J. Yin, Q. Wei, C. Wang, X. Xu, Moss-like nickel-cobalt phosphide nanostructures for highly flexible all-solid-state hybrid supercapacitors with excellent electrochemical performances, Appl. Mater. Today. 20 (2020) 100713.

[20] F. Zhao, Y. Wang, X. Xu, Y. Liu, R. Song, G. Lu, Y. Li, Cobalt Hexacyanoferrate Nanoparticles as a High-Rate and Ultra-Stable Supercapacitor Electrode Material, ACS Appl. Mater. Interfaces. 6 (2014) 11007–11012.

[21] Y. Zhang, C. Wang, X. Chen, X. Dong, C. Meng, C. Huang, Bamboo Leaves as Sustainable Sources for the Preparation of Amorphous Carbon/Iron Silicate Anode and Nickel–Cobalt Silicate Cathode Materials for Hybrid Supercapacitors, ACS Appl. Energy Mater. 4 (2021) 9328–9340.

[22] C. Xu, W. Yang, J. Zhao, J. Ma, M. Wu, Designing Multifunctional Co and Fe Co-Doped MoS2 Nanocube Electrodes for Dye-Sensitized Solar Cells, Perovskite Solar Cells, and a Supercapacitor, ACS Omega. 6 (2021) 24931–24939.

[23] K.V. Sankar, R.K. Selvan, D. Meyrick, Electrochemical performances of CoFe2O4 nanoparticles and a rGO based asymmetric supercapacitor, RSC Adv. 5 (2015) 99959–99967.

[24] X. Chen, M. Cheng, D. Chen, R. Wang, Shape-Controlled Synthesis of Co2P Nanostructures and Their Application in Supercapacitors, ACS Appl. Mater. Interfaces. 8 (2016) 3892–3900.
